# Therapy, Safety, and Logistics of Preoperative vs Postoperative Stereotactic Radiation Therapy: A Preliminary Analysis of a Randomized Clinical Trial

**DOI:** 10.1001/jamaoncol.2025.1770

**Published:** 2025-06-18

**Authors:** Debra Nana Yeboa, Jing Li, Ruitao Lin, Sujit S. Prabhu, Thomas H. Beckham, Kristina Woodhouse, Todd Allen Swanson, Jeffrey S. Weinberg, Xuemei Wang, Xiaohan Chi, Chinenye Lynette Ejezie, Dima Suki, Chenyang Wang, Chibawanye Ene, Ian E. McCutcheon, Susan McGovern, Mary Frances McAleer, Martin Tom, Amol Ghia, Subha Perni, Wen Jiang, Brian De, Caroline Chung, Betty Y. S. Kim, Barbara J. O’Brien, Jason T. Huse, Jeffrey S. Wefel, Laurence Court, Hussein Tawbi, Filip Janku, Nandita Guha-Thakurta, J. Matthew Debnam, Jason Johnson, Ceylan Altintas Taslicay, Christopher Alvarez-Breckenridge, Shaan M. Raza, Amy B. Heimberger, Franco DeMonte, Robert North, Tina M. Briere, John F. de Groot, Raymond Sawaya, David Grosshans, Frederick F. Lang, Ganesh Rao, Sherise D. Ferguson

**Affiliations:** 1 University of Texas MD Anderson Cancer Center, Houston; 2 Merck & Co, Upper Gywnedd, Pennsylvania; 3 Towson University, Towson, Maryland; 4 Monte Rosa Therapeutics, Boston, Massachusetts; 5 Yale School of Medicine, New Haven, Connecticut; 6 Northwestern University, Evanston, Illinois; 7 University of California, San Francisco; 8 American University of Beirut, Beirut, Lebanon; 9 Baylor College of Medicine, Houston, Texas

## Abstract

**Importance:**

Preoperative stereotactic radiation therapy (SRT) vs postoperative SRT logistics and toxic effects provides clinically significant data on management outcomes.

**Objective:**

To determine preoperative SRT logistics and safety profile compared with postoperative in patients with brain metastases.

**Design, Setting, and Participants:**

This single-institution phase 3 randomized clinical trial included patients 18 years and older and undergoing a planned surgical resection. Patients were required to have an Eastern Cooperative Oncology Group Performance Status score of 2 or greater and be candidates for SRT within 30 days of surgical resection. Patients with radiosensitive histologies (eg, small cell lung cancer and lymphoma), brain metastasis of unknown primary, and/or radiographic evidence of leptomeningeal disease were excluded. Data were collected from December 2018 to August 2023, and data were analyzed from September 2023 to December 2024.

**Interventions:**

Patients were randomized 1:1. Patients randomized to the preoperative SRT cohort underwent SRT (in 1 to 5 fractions) followed by surgical resection within 1 month of radiation therapy. Patients randomized to the postoperative SRT cohort underwent resection followed by postoperative SRT within 1 month of surgery.

**Main Outcomes and Measures:**

Outcomes reported focus on nonprimary end point analysis of the trial, including comparative toxic effect outcomes of preoperative vs postoperative SRT postprocedural events, feasibility of preoperative SRT, and radiation therapy management.

**Results:**

Of 103 patients, 56 (54.4%) were male, and the median (range) age was 59 (26-83) years. Of 103 patients, 83 (80.6%) completed both radiation and surgery for brain metastases while in the study. Of these, 70 patients (84%) had 1 to 4 brain metastases at enrollment, 11 (13%) had 5 to 10 lesions, and 2 (2%) had more than 10 lesions. In the preoperative stereotactic radiosurgery (SRS)/SRT cohort, 45 (88%) completed both treatments compared with 38 (73%) in the postoperative SRS/SRT arm. There were no statistically significant differences between treatment groups in 30-day postoperative morbidity or postprocedural events. The median (range) time between surgery and SRT was significantly shorter in the preoperative arm (6 [0-24] days) compared with the postoperative arm (22 [12-42] days; *P* < .001). The median (range) time from randomization to receiving both brain-directed therapies was 10 (4-31) days in the preoperative arm compared with 32.5 (19-55) days for the postoperative arm (*P* < .001).

**Conclusions and Relevance:**

In this randomized clinical trial, preoperative SRT had comparable safety to postoperative SRT and resulted in shorter time to treatment completion, potentially facilitating expedited care.

**Trial Registration:**

ClinicalTrials.gov Identifier: NCT03741673

## Key Points

**Question:**

What is the comparative clinical feasibility and safety of preoperative stereotactic radiation therapy (SRT) in a cohort of patients with surgically resectable brain metastases?

**Finding:**

In this randomized clinical trial reporting on the initial 103 patients with resectable brain metastases, preoperative SRT was not associated with increased neurological complications, and the time between surgery and radiation therapy was shorter in patients randomized to preoperative SRT, highlighting its safety and feasibility.

**Meaning:**

Preoperative SRT was safe and logistically feasible with the potential benefit of expediting treatment.

## Teaser

This randomized clinical trial evaluates preoperative stereotactic radiation therapy logistics and safety profile compared with postoperative stereotactic radiation therapy in patients with brain metastases.

## Introduction

The oncologic benefit of postoperative stereotactic radiosurgery (SRS)/stereotactic radiation therapy (SRS/SRT) following surgical resection of brain metastasis is well established. Overall, these trials demonstrated that postoperative SRS is associated with high rates of local control with minimum adverse effects, while avoiding the cognitive adverse effects of traditional whole-brain radiation therapy.^1,2,3,4,5^ Even with the success of postoperative SRS, patients with brain metastases are still susceptible to distant recurrence outside the surgical cavity, including leptomeningeal disease (LMD), a devastating subset of metastatic central nervous system disease with a dismal prognosis.^6^ Previous experience from phase 3 clinical trials of postoperative SRS reported a notable incidence of LMD. Specifically, Mahajan et al^5^ reported 12-month LMD incidence of 28% in patients receiving standard postoperative SRS. Additionally, a large retrospective review of 500 postoperative surgical cavities treated with SRS reported a 12-month LMD rate of 15.8%.^3^ This result has been reproduced in multiple follow-up studies, and overall, the reported rate of LMD in patients receiving postoperative SRS is approximately 12% to 35%.^3,5,7,8,9,10^

Preoperative SRS/SRT has been a proposed treatment strategy for resectable brain metastasis to address the challenge of leptomeningeal recurrence. Furthermore, preoperative SRS/SRT potentially offers additional radiation treatment planning and logistical benefits.^11,12,13^ There are currently several retrospective and prospective studies of preoperative SRT^8,14,15^; however, to our knowledge, there are no published randomized clinical studies evaluating the outcomes of preoperative vs postoperative SRT. Reporting the logistical operative outcomes and therapeutic management differences are important factors that are independent of the primary outcomes of local or distant intracranial recurrence. We are conducting a phase 3 clinical trial to evaluate the primary end point of the rate of LMD-free survival between patients who were randomized to receive preoperative vs postoperative SRT for surgical brain metastasis. Primary end point oncological outcomes cannot be reported in the data safety monitoring board–monitored ongoing trial at this juncture. However, in this report, we present our initial experience regarding the differences in therapy logistics of preoperative vs postoperative SRS/SRT and early safety outcomes for each treatment strategy, which significantly inform the current practice of these approaches in a clinical setting.

## Methods

### Study Design and Participants

This study was designed as a single-center phase 3 randomized clinical trial comparing preoperative and postoperative SRS/SRT for patients with brain metastases dispositioned for surgical resection. The primary objective was to investigate the 1-year LMD-free rate among patients with surgically resectable metastatic brain lesions randomized to postoperative SRT (standard care) vs preoperative SRT followed by surgery (experimental arm). The secondary objectives were to investigate the local control, distant brain metastasis rate, and overall survival. Exploratory objectives included neurocognitive function, symptom burden, health outcomes toxic effects, local control of nontarget/noncavity lesions from time of initial treatment, radiographic radiation treatment effects, biomarker tissue analysis, and cerebral spinal fluid analysis. This study protocol was approved by the MD Anderson Cancer Center Institutional Review Board and can be found in Supplement 1. All patients provided written informed consent. This study followed the Consolidated Standards of Reporting Trials (CONSORT) reporting guideline for enrolled patients with brain metastases.

As this is an ongoing data safety monitoring board–controlled study with less than 100% accrual complete, we are unable to report on primary outcomes in this report. We are reporting on initial preoperative and postoperative surgical outcomes, radiation treatment management, and logistical clinical considerations, which are highly informative to the clinical management of preoperative SRT vs postoperative SRT in the first more than 100 patients with brain metastases enrolled in the trial. For this study, we included patients 18 years and older diagnosed with brain metastases undergoing a planned surgical resection. Race and ethnicity were self-reported and documented from the electronic health record. Patients were required to have a Karnofsky performance status of at least 70 or Eastern Cooperative Oncology Group Performance Status score of 2 or more for enrollment and had to be considered candidates for SRS/SRT within 30 days of surgical resection. Patients with radiosensitive pathologies (ie, small cell lung cancer, lymphoma, multiple myeloma) or brain metastasis of unknown primary (ie, unidentifiable primary malignancy) were not eligible for enrollment. Additionally, patients with radiographic evidence of LMD (on contrast-enhanced brain or spine magnetic resonance imaging [MRI]) were also excluded.

### Procedures and Interventions

Patients randomized to the preoperative SRT cohort underwent SRT in 1 to 5 fractions followed by surgical resection within 30 days of radiation therapy. Patients randomized to postoperative SRT cohort underwent surgical resection followed by postoperative SRT within 30 days of surgery. The technique of SRS/SRT was at the discretion of the treating physician (ie, framed vs frameless, radiation modality). Target lesions were defined as the planned resected lesion that was randomized to either preoperative or postoperative SRS/SRT. Nonrandomized/nontarget lesions were unresected lesions being treated with radiation in the same therapeutic course of management. Data are presented by per-patient characteristics and by per-lesion characteristics. Time to therapy data presented per lesion reflect the time from randomization to the earliest treatment initiation of the target lesion(s) in each arm. Radiation dosing followed recommended institutional standard departmental treatment guidelines.

### Statistical Analysis

Randomization was 1:1 using the Pocock-Simon method to balance the stratification factors of target size (3 cm or smaller vs larger than 3 cm), anatomical location (supratentorial or infratentorial), and histology (ie, histologies with higher risk of LMD based on data from the prior postoperative phase 3 trial,^5^ such as breast cancer, non–small cell lung cancer, and melanoma vs others). We based our sample size justification on a log-rank test comparing freedom from LMD between groups with assumed 1-year rates of 90% vs 70% with a 2-sided 5% alpha and 80% power, accrual rate based on the prior postoperative SRS trial (2 per month), and 12-month postaccrual follow-up (ie, follow-up on all patients after last patient is accrued), requiring 110 evaluable patients. The final analysis will be performed after 22 LMD events have been observed. Taking into consideration updated dropout rates from the COVID-19 pandemic period, we plan a total accrual of up to 180 patients. This report presents preliminary analysis of the initial 103 enrolled patients with pathologically confirmed brain metastases, reporting descriptive statistics on patient characteristics, including demographic characteristics, histology, number of lesion, cavities treated, and radiation therapy. Frequency and percentages between the 2 arms on the presenting presurgical symptoms of the population enrolled and the postsurgery morbidity are reported and are compared using the χ^2^ test or Fisher exact test, as appropriate. Time to treatment, calculated from randomization, is compared between arms using the Mann-Whitney *U* test. A *P* value of less than .05 from a 2-sided test is considered statistically significant. Data analyses were performed using R version 4.3.2 (The R Foundation). Data were collected from December 2018 to August 2023, and data were analyzed from September 2023 to December 2024.

## Results

### Patient Characteristics

From December 2018 to January 2023, a total of 103 patients with pathologically confirmed brain metastases were enrolled at a single institution (Figure). The median (range) age was 59 (26-83) years, and 56 (54.4%) were male. The most common cancer diagnoses included non–small cell lung cancer (30 [29.1%]), renal cell carcinoma (15 [14.6%]), melanoma (14 [13.6%]), and breast cancer (12 [11.7%]). At baseline enrollment MRI, 86 patients had 1 to 4 brain metastases, 15 patients had 5 to 10 metastases, and 2 had more than 10 brain metastases (Table 1). Most patients (90 [87.4%]) had a single randomized target/resected lesion, and 13 (12.6%) had multiple lesions randomized. If 2 proximal/adjoining lesions were resected together and created a single cavity, they were categorized as a single cavity or target because, from a radiation dosing approach, they would be clinically treated as a single large lesion. If any 1 site had residual disease, the case was categorized as residual disease. A total of 52 patients (50.5%) had a resection for a lesion that was 3 cm or smaller. Most patients (87 [84.5%]) had supratentorial lesion(s) and 57 (55.3%) had histologies presumed to be higher risk for LMD. There were no statistical differences in the enrollment between the preoperative (n = 51) vs postoperative (n = 52) cohorts based on the above strata. Ten specific preoperative/procedural symptoms were evaluated between the treatment groups among patients who completed all protocol therapy (eTable 1 in Supplement 2). Only preoperative memory issues and unsteady gait differed between treatment cohorts. Specifically, preoperative memory deficits were observed in 1 patient (2%) in the preoperative SRT group vs 8 (21%) in the postoperative SRT group (*P* = .01). Preoperative unsteady gait was more frequent in the postoperative SRT cohort (14 [37%]) compared with the preoperative SRT cohort (6 [13%]) (*P* = .02). Data regarding the battery of neurocognitive functional baseline testing on trial and changes over time will be reported separately as a distinct end point of the study.

**Figure.**
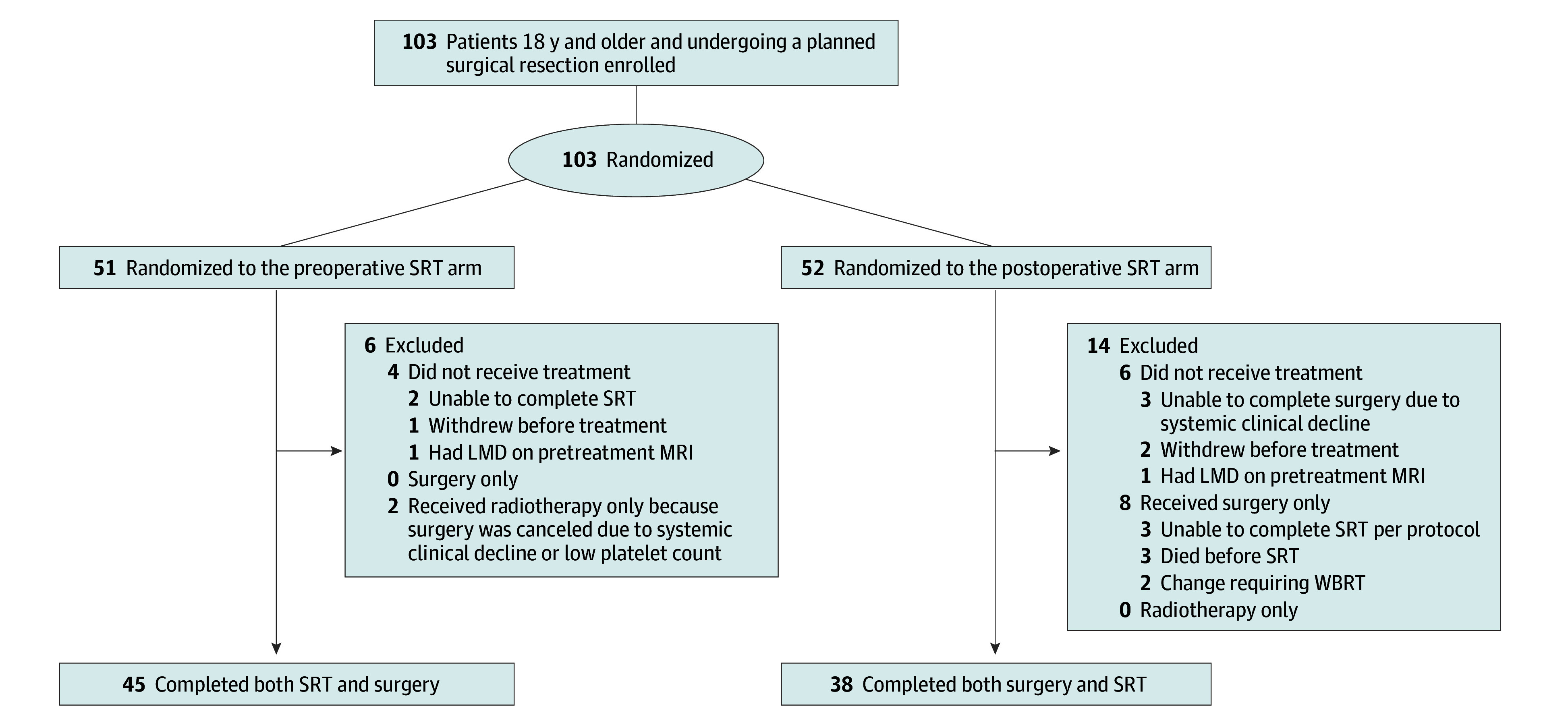
CONSORT Diagram of Brain Metastases Patients Enrolled LMD indicates leptomeningeal disease; MRI, magnetic resonance imaging; SRT, stereotactic radiation therapy; WBRT, whole-brain radiation therapy.

**Table 1.** Descriptive Statistics of Patients Enrolled in the Preoperative and Postoperative Arms

Characteristic	Patients, No. (%)		
	Total (N = 103)	Preoperative arm (n = 51)	Postoperative arm (n = 52)
Sex			
Female	47 (45.6)	25 (49.0)	22 (42.3)
Male	56 (54.4)	26 (51.0)	30 (57.7)
Age, median (range), y	59 (26-83)	61 (26-79)	59 (34-83)
Race and ethnicity^a^			
Hispanic	17 (16.5)	9 (17.6)	8 (15.4)
Non-Hispanic Black	5 (4.9)	2 (3.9)	3 (5.8)
Non-Hispanic White	75 (72.8)	36 (70.6)	39 (75.0)
Non-Hispanic other race	6 (5.8)	4 (7.8)	2 (3.8)
Treatment^b^			
No treatment	9 (8.7)	4 (7.8)	5 (9.6)
Received SRS/SRT	85 (82.5)	47 (92.1)	38 (73.1)
Received surgery	92 (89.3)	45 (88.2)	47 (90.4)
Received SRS/SRT and surgery	83 (80.6)	45 (88.2)	38 (73.1)
Primary histology			
Breast	12 (11.7)	6 (11.8)	6 (11.5)
Melanoma	14 (13.6)	8 (15.7)	6 (11.5)
NSCLC	30 (29.1)	13 (25.5)	17 (32.7)
Renal cell carcinoma	15 (14.6)	8 (15.7)	7 (13.5)
Other	32 (31.0)	16 (31.3)	16 (30.8)
Resected lesions			
1/1^c^	90 (87.4)	46 (90.2)	44 (84.6)
>1	13 (12.6)	5 (9.8)	8 (15.4)
Brain lesions at baseline			
1-4	86 (83.5)	43 (84.3)	43 (82.7)
5-10	15 (14.6)	7 (13.7)	8 (15.4)
>10	2 (1.9)	1 (2.0)	1 (1.9)
Stratification factors			
Maximum size of brain lesion			
≤3 cm	52 (50.5)	24 (47.1)	28 (53.8)
>3 cm	51 (49.5)	27 (52.9)	24 (46.2)
Location			
Supratentorial	87 (84.5)	43 (84.3)	44 (84.6)
Infratentorial	16 (15.5)	8 (15.7)	8 (15.4)
Histology			
Histology with presumed higher risk of LMD (ie, breast, NSCLC, melanoma)	57 (55.3)	28 (54.9)	29 (55.8)
Other histology	46 (44.7)	23 (45.1)	23 (44.2)

Abbreviations: LMD, leptomeningeal disease; NSCLC, non–small cell lung cancer; SRS, stereotactic radiosurgery; SRT, stereotactic radiation therapy.

^a^ Race and ethnicity were self-reported and documented from the electronic health record. The other race category included Asian race and those self-identifying as other race.

^b^ Categories are not mutually exclusive, and column percentages exceed 100%.

^c^ Two proximal/adjoining lesions resected together creating a single cavity.

### Treatment of Enrolled Patients

#### Patient Characteristics

Of 103 enrolled patients (Figure), 83 (80.6%) completed both radiation and surgery for brain metastases (Table 2). Approximately 10% did not receive therapy after enrollment. The distribution by histology was similar between the 2 randomized groups. Patients’ baseline MRI were evaluated at time of enrollment. Of those completing protocol therapy, 70 (84%) had 1 to 4 brain metastases at enrollment, 11 (13%) had 5 to 10 lesions, and 2 (2%) had more than 10 lesions. Overall, there were slight shifts in the number of total lesions requiring radiation at the time of treatment compared with baseline, with more patients having 5 to 10 lesions by time of therapy, with a median (range) brain metastasis increase of 2 (1-8). eTable 2 in Supplement 2 includes resection characteristics.

**Table 2.** Descriptive Statistics of Patients Enrolled in the Preoperative vs Postoperative Trial Who Completed Both Therapies

Characteristic	Patients, No. (%)		
	Total (n = 83)	Preoperative arm (n = 45)	Postoperative arm (n = 38)
Sex			
Female	39 (47.0)	21 (46.7)	18 (47.4)
Male	44 (53.0)	24 (53.3)	20 (52.6)
Age, median (range), y	59 (26-83)	61 (26-79)	59 (37-83)
Race and ethnicity^a^			
Hispanic	14 (16.9)	8 (17.8)	6 (15.8)
Non-Hispanic Black	4 (4.8)	2 (4.4)	2 (5.2)
Non-Hispanic White	59 (71.1)	31 (68.9)	28 (73.7)
Non-Hispanic other race	6 (7.2)	4 (8.9)	2 (5.2)
Primary histology			
Breast	8 (9.6)	4 (8.9)	4 (10.5)
Melanoma	10 (12.0)	6 (13.3)	4 (10.5)
NSCLC	27 (32.5)	13 (28.9)	14 (36.8)
Renal cell carcinoma	15 (18.1)	8 (17.8)	7 (18.4)
Other	23 (27.7)	14 (31.1)	9 (23.7)
Resected lesions			
1/1^b^	73 (88.0)	41 (91.1)	32 (84.2)
>1	10 (12.0)	4 (8.9)	6 (15.8)
Brain lesions at baseline			
1-4	70 (84.3)	38 (84.4)	32 (84.2)
5-10	11 (13.3)	6 (13.3)	5 (13.2)
>10	2 (2.4)	1 (2.2)	1 (2.6)
Total treated lesions at time of radiation			
1-4	67 (80.7)	39 (86.7)	28 (73.7)
5-10	14 (16.9)	5 (11.1)	9 (23.7)
>10	2 (2.4)	1 (2.2)	1 (2.6)
Total treated nonresected lesions			
0	33 (39.8)	16 (35.6)	17 (44.7)
1-4	40 (48.2)	25 (55.6)	15 (39.5)
5-10	8 (9.6)	3 (6.7)	5 (13.2)
>10	2 (2.4)	1 (2.2)	1 (2.6)
Treatment of resected lesion			
GK only	57 (68.7)	38 (84.4)	19 (50.0)
Linear accelerator only	26 (31.3)	7 (15.6)	19 (50.0)
Fractionation of planned resected/target lesion			
1 Fraction only	34 (41.0)	23 (51.1)	11 (28.9)
3-5 Fractions only	47 (56.6)	21 (46.7)	26 (64.8)
Both 1 and 3-5 fractions	2 (2.4)	1 (2.2)	1 (2.6)
Treatment of nonresected/nontarget lesion			
GK only	48 (57.8)	27 (60.0)	21 (55.3)
Linear accelerator only	1 (1.2)	1 (2.2)	0
Both GK and linear accelerator	1 (1.2)	1 (2.2)	0
Fractionation of nonresected/nontarget lesion			
1 Fraction only	45 (54.2)	27 (60.0)	18 (47.4)
3-5 Fractions only	1 (1.2)	1 (2.2)	0
Both 1 and 3-5 fractions	4 (4.8)	1 (2.2)	3 (7.9)

Abbreviations: GK, Gamma Knife; NSCLC, non–small cell lung cancer.

^a^ Race and ethnicity were self-reported and documented from the electronic health record. The other race category included Asian race and those self-identifying as other race.

^b^ Two proximal/adjoining lesions resected together creating a single cavity.

#### Radiation Therapy

For preoperative or postoperative SRT, the choice of machine modality was at the discretion of the treating physician reflecting the real-world setting of different machinery techniques available nationally for standard treatment. Typically, only 1 modality was used for all targets (lesion or cavity). At our institution, 57 patients (69%) were treated with Gamma Knife and 26 (31%) were treated with linear accelerator. Most patients randomized to the preoperative cohort (38 [84%]) received Gamma Knife compared with 19 patients in the postoperative group (50%) (*P* = .002). Patients treated with 1 fraction represented 51% of the preoperative group (n = 23) and 29% of the postoperative group (n = 11) (Table 2). Overall, the most common radiation dose range for single-fraction SRS was 17 to 19 Gy (27 [29%]; most received 18 Gy). The target lesion volume necessitated this dose range in 19 in the preoperative group (38%) and 8 in the postoperative group (19%). For multifraction SRT, the most common prescription dose range was 24 to 27 Gy in 3 fractions (48 [52%]; most received 27 Gy). This dose was more common in the postoperative arm group (28 [65%]) compared with the preoperative SRT group (20 [40%]). A total of 148 nonresected lesions were treated. The most common single-fraction prescription dose was 20 to 22 Gy (137 [92.6%]) followed by 17 to 18 Gy (6 [4.1%]). The most common multifraction SRT dose for nontarget/randomized cavity lesions was 24 to 27 Gy in 3 fractions in both treatment groups (Table 3). There were no statistical differences in the RT dose ranges between the 2 randomized groups.

**Table 3.** Per-Lesion Tumor Characteristics and Time to Therapy

Characteristic	Lesions, No. (%)			*P* value
	Total	Preoperative arm	Postoperative arm	
Cavities treated per protocol, No.	94	50	44	NA
Anatomic lobe				
Frontal	40 (42.6)	18 (36.0)	22 (50.0)	.04
Parietal	23 (24.5)	9 (18.0)	14 (31.8)	
Temporal	11 (11.7)	7 (14.0)	4 (9.1)	
Occipital	9 (9.6)	8 (16.0)	1 (2.3)	
Cerebellar	11 (11.7)	8 (16.0)	3 (6.8)	
Planned treatment dose resected lesion				
Single-fraction targets	38 (40.9)	26 (52.0)	12 (27.9)	
15-16 Gy	5 (5.4)	4 (8.0)	1 (2.3)	.55
17-19 Gy	27 (29.0)	19 (38.0)	8 (18.6)	
20-22 Gy	6 (6.5)	3 (6.0)	3 (7.0)	
Multifraction targets	54 (58.1)	23 (46.0)	31 (72.1)	
21-23 Gy in 3 fractions	2 (2.2)	0	2 (4.7)	.27
24-27 Gy in 3 fractions	48 (51.6)	20 (40.0)	28 (65.1)	
25-30 Gy in 5 fractions	4 (4.3)	3 (6.0)	1 (2.3)	
Nontarget lesions treated per protocol, No.	148	72	76	NA
Planned treatment dose nontarget lesion				
Single-fraction lesions	143 (96.6)	70 (97.2)	73 (96.1)	
15-16 Gy	0	0	0	>.99
17-19 Gy	6 (4.1)	3 (4.2)	3 (3.9)	
20-22 Gy	137 (92.6)	67 (93.1)	70 (92.1)	
Multifraction lesions	5 (3.4)	2 (2.8)	3 (3.9)	
24-27 Gy in 3 fractions	5 (3.4)	2 (2.8)	3 (3.9)	>.99
25-30 Gy in 5 fractions	0	0	0	
Time to therapy, median (range), d				
Time from randomization to start of RT	13.0 (0-55)	6.0 (0-14)	32.5 (19-55)	<.001
Time from randomization to surgery	9.0 (1-31)	10.0 (4-31)	8.5 (1-29)	.06
Time between start of RT and surgery	14.0 (0-42)	6.0 (0-24)	22.0 (12-42)	<.001
Time from randomization to receipt of both RT and surgery	21.0 (4-55)	10.0 (4-31)	32.5 (19-55)	<.001

Abbreviations: NA, not applicable; RT, radiation therapy.

#### Timing of Therapy

Among the patients enrolled in the preoperative SRT cohort, 45 (88%) completed both treatments compared with 38 (73%) in the postoperative SRT arm (*P* = .09). The time between treatment interventions (surgery and SRT) was shorter in the preoperative arm. Specifically, in the preoperative arm, the median (range) time between SRT (first intervention) and surgery (second intervention) was 6 (0-24) days. Conversely, in the postoperative arm, the timing between surgery (first intervention) and SRT (second intervention) was 22 (12-42) days (*P* < .001). The median (range) time from randomization to RT was 6 (0-14) days in the preoperative arm vs 32.5 (19-55) days in the postoperative arm (*P* < .001). The median (range) time from randomization to surgery was 10 (4-31) days in the preoperative arm compared with 8.5 (1-29) days in the postoperative arm (*P* = .06), indicating no significant delay in planned surgery. The median (range) time from randomization to receiving both RT and surgery was significantly shorter in the preoperative arm (10 [4-31] days) compared with the postoperative arm (32.5 [19-55] days; *P* < .001).

### Postprocedural Events in Enrolled Patients

Thirty-day postoperative morbidity (or events) between the 2 randomized groups were not statistically significant (Table 4). Specifically, 22 postoperative events were reported in the preoperative SRT group vs 17 events in the postoperative SRT group (*P* = .87). Event types included neurological, respiratory, infectious, vascular, cardiac, gastrointestinal, or genitourinary events and minor events. The 30-day surgical postoperative event reporting is separate from the final total adverse event analysis, which must be reserved to the conclusion of this study.

**Table 4.** Descriptive Statistics of Postsurgical Procedure 30-Day Events by Randomized Group

Event	Patients, No. (%)			*P* value
	Total (n = 83)	Preoperative arm (n = 45)	Postoperative arm (n = 38)	
Any 30-d postoperative event	39 (47.0)	22 (48.9)	17 (44.7)	.87
Event by organ system				
Neurological event				
Any neurological	30 (36.1)	16 (35.5)	14 (36.8)	.70
Focal motor deficit	12 (14.5)	7 (15.6)	5 (13.2)	>.99
Focal sensory deficit	4 (4.8)	2 (4.4)	2 (5.3)	>.99
Speech impairment	0	0	0	>.99
Visual impairment	3 (3.6)	0	3 (7.9)	.07
Cranial nerve deficit	0	0	0	>.99
Hydrocephalus	0	0	0	>.99
Radiation necrosis	0	0	0	>.99
Progressive edema	0	0	0	>.99
Cerebral abscess	0	0	0	>.99
Seizures	2 (2.4)	2 (4.4)	0	.49
CSF leakage	0	0	0	>.99
Pneumocephalus	1 (1.2)	1 (2.2)	0	>.99
Hemorrhage	0	0	0	>.99
Intracranial	0	0	0	>.99
Subdural	0	0	0	>.99
Epidural	0	0	0	>.99
Intraparenchymal	0	0	0	>.99
Other neurological^a^	23 (27.7)	13 (28.9)	10 (26.3)	>.99
Respiratory event				
Any respiratory	8 (9.6)	4 (8.9)	4 (10.5)	.70
Respiratory failure	1 (1.2)	0	1 (2.6)	.43
Atelectasis	0	0	0	>.99
Pneumothorax	0	0	0	>.99
Pulmonary embolus	4 (4.8)	3 (6.7)	1 (2.6)	.61
Other respiratory	5 (6.0)	1 (2.2)	4 (10.5)	.14
Infectious disease event				
Any infectious disease	5 (6.0)	2 (4.4)	3 (7.9)	.63
Wound	1 (1.2)	0	1 (2.6)	.43
Meningitis	0	0	0	>.99
Encephalitis	0	0	0	>.99
Pneumonia	2 (5.1)	1 (2.2)	1 (2.6)	>.99
Bacteremia	0	0	0	>.99
UTI	2 (2.4)	1 (2.2)	1 (2.6)	>.99
Other infectious disease	1 (1.2)	0	1 (2.6)	.43
Peripheral/vascular event				
Any peripheral/vascular	0	0	0	>.99
DVT	0	0	0	>.99
Other peripheral/vascular	0	0	0	>.99
GI tract event				>.99
Any GI tract	3 (3.6)	2 (4.4)	1 (2.6)	>.99
Bleed	0	0	0	>.99
Perforation	0	0	0	>.99
GI tract obstruction	0	0	0	>.99
Other GI tract	3 (3.6)	2 (4.4)	1 (2.6)	>.99
Genitourinary event				
Any genitourinary	4 (4.8)	2 (4.4)	2 (5.3)	>.99
Retention	1 (1.2)	1 (2.2)	0	>.99
Incontinence	1 (1.2)	1 (2.2)	0	>.99
Other genitourinary	3 (3.6)	1 (2.2)	2 (5.3)	.57
Cardiac event				
Any cardiac	8 (9.6)	3 (6.7)	5 (13.2)	.26
Myocardial infarction	0	0	0	>.99
Transischemia	0	0	0	>.99
Arrythmia	1 (1.2)	0	1 (2.6)	.43
Other cardiac	6 (7.2)	3 (6.7)	3 (7.9)	>.99
Angiogram	1 (1.2)	0	1 (2.6)	.43

Abbreviations: CSF, cerebrospinal fluid; DVT, deep vein thrombosis; GI, gastrointestinal; UTI, urinary tract infection.

^a^ May include nonspecific tremors, insomnia, agitation or behavioral changes, or difficulty concentrating.

## Discussion

To address the potential benefit of preoperative SRT, we are conducting a phase 3 single-institution trial randomizing patients with surgically resectable brain metastasis to receive either preoperative or postoperative SRT. In this report, we evaluated the initial subset population of patients with surgical brain metastases treated preoperatively or postoperatively with radiation therapy in this study and report the logistics and safety. Although we cannot yet report the primary end point oncologic outcomes of the treatment cohorts, our presented data are highly relevant to clinicians using neoadjuvant SRT regarding expected feasibility, logistics, and appropriate target populations.

A powerful advantage of preoperative SRT delivery is the logistical aspects, specifically the higher likelihood of patient completion of both surgery and radiation therapy and decreased treatment delay. Unexpected events and/or complications can extend postoperative treatment delay. The metastatic cancer population is often older and may have additional medical comorbidities complicating their postoperative course and recovery and ultimately delay adjuvant therapy. The cumulative impact of these logistical issues is highlighted in a phase 2 clinical trial by Brennan at al,^16^ focused on postoperative SRS. In this trial, they found that 20% of postoperative patients did not undergo their scheduled adjuvant SRS.^16^ These may also have implications for the delay of SRT impacting oncology outcomes. In our initial observation, we found that a higher proportion of patients in the preoperative SRS group completed their treatment course (surgery plus SRT) compared with the postoperative SRT cohort (45 [88%] vs 38 [73%]).

In addition to adherence, there is also the issue of timing. For postoperative SRT, the patient is typically required to wait 10 to 14 days to allow for incision healing. This also allows time for resolution of immediate radiographic postoperative changes and surgical cavity size stabilization. This wait time is not necessary with preoperative treatment, and there is an opportunity to increase treatment efficiency. Preoperative SRT and surgery can be completed within the same week and, if carefully planned, potentially the same hospitalization. Expedited radiation therapy may reflect an interest in not delaying surgery for lesions that are infratentorial or with radiographic compressive findings. Furthermore, time between surgical resection and adjuvant SRS has been associated with rates of local recurrence, with a median time to postoperative SRS of 37 days associated with a rate of 16%.^17^ Thus far in our trial, the time to completion of all therapies (surgery and SRT) was approximately 3-fold longer in the postoperative SRT group compared with the preoperative SRT group. In addition to improved patient satisfaction, completing cranial treatment quickly facilitates expedited initiation of systemic therapy, which is critical in this population with advanced metastatic disease.

One of the concerns regarding preoperative SRT is upfront radiation to lesions that are often large and/or symptomatic. Importantly, we did not observe a significant difference between treatment cohorts with regard to 30-day postprocedure complications. Our observation is consistent with the current literature reporting the safety of preoperative SRT,^14,18^ although those reports were not phase 3 randomized clinical trials. Another concern from patients and clinicians has been that awaiting preoperative SRT may significantly delay a patient’s surgery because the logistics for RT may require additional planning time upfront. Our analysis found that the time to completion of surgery was not statistically different between the 2 groups (median [range] time, 10 [4-31] days in the preoperative arm compared with 8.5 [1-29] days in the postoperative arm).

Lastly, the treatment algorithm of preoperative SRT has potential advantages in treatment planning and addressing the concern of intraoperative tumor spillage. It has been suggested that surgical resection itself may disrupt anatomical borders, theoretically contributing to cerebral spinal fluid contamination with malignant cells and increased risk of LMD.^19^ One potential benefit of neoadjuvant treatment is to treat the tumor and cavity prior to surgical manipulation. Thus, tumor cells fated to spill into the resection cavity have already been treated with SRT and thus are less likely to recur locally or proliferate distantly and establish LMD.^8^ In addition, the treatment planning benefits of preoperative SRT are also discussed in the literature due to improved target delineation.^11,12,13,20^ Our data highlight how radiation treatment planning has been comparable in both groups despite the target tumor size.

### Limitations

Our initial findings on surgical outcomes and treatment logistics have limitations as a subset cohort analysis of an ongoing trial. Our preliminary analysis represents 30-day surgical outcomes, and long-term follow-up is of value. While final reporting of trial analysis and adverse events could theoretically reveal additional insights, our current large sample size of more than 100 patients may mitigate that likelihood. Next, the study is potentially limited by therapy reflecting a high-volume academic institution with the capacity to deliver SRT to acute inpatients, which may not be feasible at all institutions. This may result in greater trial enrollment of patients with larger volume brain metastases compared with community practice of SRT. Trial participation is at the discretion of the clinical team, and thereby patients that are emergent or have lesions deemed neurosurgically high risk, such as patients requiring intensive care unit therapy or lesions that are 5.5 cm or larger, were often not enrolled. Lastly, we did not enroll patients with unknown primary diagnosis, and our data would not apply to this population.

## Conclusions

In conclusion, our current findings of this randomized clinical trial are significant to the real-world community offering preoperative or postoperative SRS/SRT and highlight minimal differences in postoperative 30-day morbidity. It also provides unique insights from other currently enrolling clinical trials that may differ in approach and methodology. For instance, our study provides analysis of both single-fraction SRS and multifractionation SRT, which is applicable to more real-world radiation oncology practices. We report in one of the earliest phase 3 randomized clinical trials that preoperative SRT is logistically feasible and potentially favorable compared with postoperative SRT, with more patients completing the planned treatment course (radiation and surgery) and with shorter time to completion of brain-directed therapy.

## Notes

Supplement 1. Trial Protocol

Supplement 2. **eTable 1.** Presurgical Procedure Signs or Symptoms by Randomized Group of Preoperative Radiation vs Postoperative Radiation **eTable 2.** Description of SRT, Radiographic, and Surgical Characteristics of Patients With Brain Metastases Enrolled in the Preoperative vs Postoperative Trial that Completed Both Therapies

Supplement 3. Data Sharing Statement
