# Supplementary material for: Therapy, Safety, and Logistics of Preoperative vs Postoperative Stereotactic Radiation Therapy: A Preliminary Analysis of a Randomized Clinical Trial
Source: JAMA Oncol. 2025 Jun 18;11(8):890–9. doi: 10.1001/jamaoncol.2025.1770 (PMC12177721; doi:10.1001/jamaoncol.2025.1770)
Supplement: Supplement 1. — Trial Protocol [file jamaoncol-e251770-s001.pdf]

TITLE: A PHASE III TRIAL OF PRE-OPERATIVE STEREOTACTIC RADIOSURGERY (SRS) VERSUS POST-OPERATIVE SRS FOR BRAIN METASTASES

LEAD PRINCIPAL INVESTIGATOR:
Debra Nana Yeboa
Radiation Oncology
713-563-2300
DNYeboa@mdanderson.org

TABLE OF CONTENTS

A1. TRIAL SUMMARY
1 OBJECTIVES
1.1 PRIMARY OBJECTIVE
1.2 SECONDARY OBJECTIVES
1.3 EXPLORATORY OBJECTIVES
2 BACKGROUND
2.1 RATIONALE
2.2 TECHNIQUE OF STEREOTACTIC RADIOSURGERY
2.3 SURGICAL MANAGEMENT OF METASTATIC BRAIN TUMORS
2.4 STEREOTACTIC RADIOSURGERY AFTER SURGICAL RESECTION
2.5 PRE-OPERATIVE STEREOTACTIC RADIOSURGERY
2.6 NEUROCOGNITIVE FUNCTION ASSESSMENT
2.7 PATIENT-REPORTED OUTCOMES (PROS) AND HEALTH-RELATED QUALITY OF LIFE (HRQOL)
3 FEASIBILITY
4 SIGNIFICANCE/ VALUE OF PROSPECTIVE TRIAL
5 PATIENT SELECTION, ELIGIBILITY, AND INELIGIBILITY CRITERIA
5.1 INCLUSION CRITERIA
5.2 EXCLUSION CRITERIA
6 REQUIREMENTS FOR STUDY ENTRY, TREATMENT, AND FOLLOW-UP
6.1 PRETREATMENT EVALUATION
6.2 STRATIFICATION
6.3 PRE-TREATMENT ASSESSMENTS
6.4 ASSESSMENTS IN FOLLOW UP

6.5 STUDY CHART
7 TREATMENT PLAN/REGIMEN DESCRIPTION
7.1 RADIATION THERAPY
7.2 DEFINITION OF TARGET VOLUMES AND MARGINS
7.3 DOSE AND DOSE CONSTRAINTS
7.4 IMMOBILIZATION AND SIMULATION
7.5 SIMULATION IMAGING
7.6 MRI TREATMENT IMAGING
7.7 PATIENT-SPECIFIC QA
7.8 DAILY TREATMENT LOCALIZATION/IGRT
8 DEFINITION FOR LEPTOMENINGEAL DISEASE
9 OFF STUDY REQUIREMENTS
10 ADVERSE EVENTS REPORTING REQUIREMENTS
11 SPECIMEN COLLECTION
12 OPTIONAL SPECIMEN SUBMISSIONS
13 RADIOMICS STUDIES (NON-TISSUE)
14 NEUROCOGNITIVE EVALUATION
14.1 NEUROCOGNITIVE CLINICAL TRIAL BATTERY
14.2 HEALTH RELATED QUALITY OF LIFE
15 STATISTICAL CONSIDERATIONS
15.1 STATISTICAL ANALYSIS/POWER/STUDY DESIGN
15.2 STRATIFICATION
15.3 RANDOMIZATION
15.4 TOTAL ACCRUAL
15.5 STUDY ENDPOINTS
15.6 SECONDARY ENDPOINTS
15.7 EXPLORATORY ENDPOINTS
15.8 PRIMARY OBJECTIVES STUDY DESIGN
15.9 DEFINITIONS OF PRIMARY ENDPOINTS AND HOW THESE WILL BE ANALYZED
15.10 ANALYSIS OF SYMPTOM BURDEN
15.11 ASSESSMENT OF QUALITY ADJUSTED SURVIVAL
15.12 ANALYSIS OF EXPLORATORY ENDPOINT/RADIOGRAPHIC EVALUATION
16 DATA COLLECTION
17 QUALITY ASSURANCE FOR CANCER NETWORK SITES

CONFIDENTIAL

A1. TRIAL SUMMARY

Abbreviated Title	Pre-op vs Post-op SRS for Brain Metastasis Phase III Trial
Trial Phase	Phase III
Clinical Indication	Brain metastasis dispositioned to surgery and SRS
Trial Type	Treatment
Type of control	Post-op SRS
Route of administration	External beam photon radiation therapy with surgery
Trial Blinding	NA
Treatment Groups	Two arms, randomized<br>Arm 1: Surgery followed by SRS<br>Arm 2: SRS followed by Surgery
Number of trial subjects	up to 180
Estimated duration of trial	67 months
Duration of Participation	2 years

1. OBJECTIVES

1.1 PRIMARY OBJECTIVE

To investigate the 1 year leptomeningeal disease (LMD)-free rate among patients with surgically resectable metastatic brain lesions randomized to post-operative stereotactic radiosurgery (SRS) (standard care) versus pre-operative SRS followed by surgery (experimental arm).

Hypotheses: We hypothesize the 1 year LMD-free rate will increase from 70% with post- operative SRS to 90% with pre- operative SRS (1,2). The outcome from this phase will be a new paradigm for the treatment of brain metastasis. It would decrease the incidence of a fatal neurological state of disease by decreasing the rate of LMD in brain metastasis patients living out to at least 1 year.

1.2 SECONDARY OBJECTIVES

To investigate the local control (LC), distant brain metastasis rate, and overall survival (OS) of pre-operative (pre-op) vs post-operative (post-op) SRS in patients with brain metastasis.

Hypotheses: We hypothesize that local control, distant brain metastasis rate, and overall survival will be equivalent between pre-operative SRS and post-operative SRS. The outcome of this phase would establish the non-inferiority of pre-operative SRS to post-operative SRS in clinical endpoints pertinent to the overall clinical management of brain metastases patients.

CONFIDENTIAL

1.3 EXPLORATORY OBJECTIVES

1.3.1 To assess the reliability of different imaging features by using a combination of patient data and phantom data to quantify the uncertainties associated with using MRI for radiomics studies. This aim includes an evaluation of the impact of the method used for feature (or pixel-value) normalization, image preprocessing, and also the impact of tumor volume from MRIs already obtained as standard of care.

Hypotheses: We hypothesize that the radionomic features with most predictive value will be the histogram of oriented gradients (HOG) on T2 weighted MRI sequences and gray level co-occurrence matrix (COM) on T1 with contrast on 3D imaging. The outcome from this phase will be a well-tested tool for MRI radiomics studies, an understanding of the reliability of different categories (or sub-categories) of image features, and lists of robust features. We will use this tool to examine treatment response in groups of patients with different diseases and imaging characteristics.

1.3.2 To assess the correlation of imaging-pathology correlates using multiparametric imaging that characterize the tumor and peri-tumoral microenvironment including features such as tumor vascular characteristics, cellular density, oxygenation, and presence of inflammation/immune reactivity.

Hypotheses: We hypothesize that measures of tumor vascularity, cellular density and oxygenation on imaging will be correlated with these features on tumor pathology. We also hypothesize that differences in these parameters will be measurable pre- and post-radiosurgery treatment. The findings from this exploratory analysis will be used to guide future investigations that aim to validate these quantitative measure with a better understanding of the underlying mechanisms reflected in the imaging parameters.

1.3.3 To investigate the neuro-cognitive impact, patient reported outcomes and health-related quality of life of pre-operative (pre-op) vs post-operative (post-op) SRS in patients with brain metastasis.

CONFIDENTIAL

Hypotheses: We hypothesize that neuro-cognition will be equivalent between pre-operative SRS and post-operative SRS. We hypothesize patient reported outcomes and health-related quality of life at 1 year will be improved with pre-operative SRS due to lower disease burden with LMD.

(Exploratory Objectives are primarily designed for UT MD Anderson Cancer Center patients.)

2. BACKGROUND

2.1 RATIONALE

At least 1 in 7 patients who undergo resection for a single brain metastasis followed by standard of care post-op radiation will subsequently develop leptomeningeal disease, a terminal and incurable state of disseminated central nervous system (CNS) metastasis. Post-operative stereotactic radiosurgery (SRS) is associated with a risk of leptomeningeal disease (LMD) of between 3% to 28% (1, 2), and most commonly an average risk of approximately 10% to 15% (3, 4). Surgical resection may be more challenging and may be associated with a higher risk of seeding and LMD including in the posterior fossa (PF) of the brain. Overall, the post-operative LMD risk may be double that of patients undergoing SRS alone which has a risk of 2 to 6%(1, 3). The role of pre-operative SRS in the prospective setting is under-explored and offers the potential to sterilize the brain tumor margins prior to resection and thus minimize the dissemination of cancer cell intraoperatively. Additionally, radiosurgical planning is easier in the preoperative setting because surgery disrupts anatomical structures making margins less certain. In retrospective series, the LMD risk may be reduced to 3.5% (5), comparable to those undergoing SRS alone. In a multi-institutional retrospective review of 180 patients receiving pre-operative SRS versus post-operative SRS, the rate of LMD for post-operative SRS was compared to the pre-operative SRS rate. The post-op SRS LMD rate at 2 years was 16.6% vs 3.2% for patients receiving pre-operative SRS.(4) Thus supporting evidence for the rationale of evaluating pre-operative SRS over post-operative SRS.

Our study will address the national knowledge gap on the role of pre-operative stereotactic radiosurgery in reducing the risk of LMD. The significance of this is decreasing the incidence of LMD in patients undergoing surgery, and importantly delaying time to whole brain radiation therapy (WBRT) avoiding deleterious neurocognitive effects associated with WBRT, and decreasing the likelihood of cause of death due to neurological disease progression. Our study proposal will randomize patients to pre-operative SRS versus post-operative SRS for patients with brain metastasis. The primary aim of our trial and proposal is determining if pre-operative

CONFIDENTIAL

SRS can increase the LMD free rate to that of post-operative SRS. The long term goal of this trial would be to decrease the overall incidence of LMD in brain metastasis and establish a new option and paradigm in care for the treatment of brain metastasis. By decreasing the incidence of LMD, our study will establish pre-operative SRS as the preferred modality for adjunctive treatment after surgical resection of brain metastases.

Secondary aims include assessing local control (LC), distant brain metastasis rate, overall survival (OS) and neuro-cognitive outcomes for pre-operative SRS in brain metastases. Tertiary correlative study aims include using MR imaging and radiomics, changes in imaging features over time, and correlation with pathology to determine response to treatment. MR images are particularly rich in information about the tumor phenotype, and MRI-based radiomics and delta-radiomics research could be effective in identifying and later predicting treatment response. Our collaborator's overall hypothesis is that quantitative imaging features, extracted from serial standard of care MRI studies and pathology, will allow us to probe the tumor phenotype, including its response to treatment, which will translate into clinical trial decision support.

2.2 TECHNIQUE OF STEREOTACTIC RADIOSURGERY

Stereotactic radiosurgery can be delivered in multiple different methods. Among these include single fraction therapy with *Perfexion* Gamma Knife, multi-fractions radiosurgery with *ICON* Gamma Knife, and multi-fraction radiosurgery with LINACs. Gamma Knife stereotactic radiosurgery is a method of eradicating intracranial lesions by delivering multiple, small, well collimated beams of ionizing radiation to stereotactically localized lesions. In 1951 at the Karolinska Institute in Sweden, Lars Leksell originally developed the technique for the treatment of functional disorders of the brain by ablating specific sites. Now with the development of MRI in the 1980s, stereotactic radiosurgery has now been shown to be effective against brain tumors. Next, more recently, technical advances in the design of conventional radiotherapy units allowed them to be used as radiosurgery units. Specifically, modern linear accelerators have the ability to generate high-energy beams of x-rays that can be well collimated. These machines can administer arc photon beams precisely around a fixed point. The LINAC-based stereotactic radiosurgery technique allows delivery of high doses of radiation to stereotactically defined targets with a steep dose gradient at the edge of the lesions and a minimal dose to the surrounding brain.

At the University of Texas MD Anderson Cancer Center (UTMDACC), LINAC-based stereotactic radiosurgery technique and Gamma Knife have been available since 1990's and our

CONFIDENTIAL

multidisciplinary team comprised of neurosurgeons, radiation oncologists and radiation physicists treat over 200 patients per year.

2.3 SURGICAL MANAGEMENT OF METASTATIC BRAIN TUMORS

Two randomized controlled trials definitively showed a benefit to surgical resection followed by whole brain radiation. In the 1990 study by Patchell et al., surgical resection followed by postoperative radiation was significantly superior to biopsy followed by radiation (local recurrence rates were 20% vs. 52 % respectively).(6) Median overall survival was superior as well (40 weeks vs. 15 weeks respectively). In a subsequent study, the same authors showed that the addition of whole brain radiation to a surgically resected single brain metastasis was superior to resection alone. In this study, the local recurrence rates were 10% versus 46% respectively.(7) At MDACC, surgical resection is frequently employed for treatment of metastatic brain disease, and we have specific expertise in this area. Our neurosurgeons make use of state of the art techniques (including frameless stereotactic navigation), and achieve complete resection of the tumor in 95% of cases.

2.4 STEREOTACTIC RADIOSURGERY AFTER SURGICAL RESECTION

The only randomized trial of post-op SRS versus observation is our institutional trial. In this study Mahajan et al identified a local control rate of 72% at 12 months using a de-escalation SRS dose to the cavity.(2) Our current study would address both pre-operative SRS as well as dosing with some similarity to the standard NC107 dosing(8) using volume of the lesion and cavity for the radiation dose. This would offer both new information on pre-operative SRS as well as cavity local control with standard dosing in a randomized controlled setting.

2.5 PRE-OPERATIVE STEREOTACTIC RADIOSURGERY

In a multi-institutional retrospective review of 180 patients receiving pre-operative SRS versus post-operative SRS, the rate of LMD for post-operative SRS was compared to the pre-operative SRS rate. The post-op SRS LMD rate at 2 years was 16.6% vs 3.2% for patients receiving pre-operative SRS. Multivariable analyses revealed no difference between groups for overall survival, local recurrence, and distant brain recurrence.(4) In another retrospective multi-institutional study, pre-operative SRS was compared to post-operative whole brain radiation therapy (WBRT). Intracranial outcomes were also similar including local recurrence, distant brain failure. With post-operative whole brain radiation therapy, the rate of LMD was reduced to 9% and comparable to that of pre-operative SRS at 3.5%, and thus not statistically significantly different. The tradeoff is

CONFIDENTIAL

that patients required WBRT to decrease the likelihood of LMD and were exposed to the deleterious neurocognitive risks associated with that modality.(5) The existing data shows the potential for pre-operative SRS to improve an important clinical outcome. However, retrospective series may be subject to selection bias and the hypothesis must be tested in a randomized controlled study to accurately define the role and extent of benefit of pre-operative SRS to the standard of post-operative SRS.

2.6 NEUROCOGNITIVE FUNCTION ASSESSMENT

The Neurocognitive Clinical Trial Battery is a brief, sensitive, repeatable, highly standardized, objective battery of neurocognitive tests that have been demonstrated to be practical in terms of burden on the patient and site, with good compliance in multicenter clinical trials.(9) The following battery of tests was utilized in CC001 (WBRT with hippocampal sparing and Memantine; Paul Brown), which serves as the basis for the current trial. Neurocognitive function will be assessed using the Hopkins Verbal Learning Test – Revised(10) (HVLT-R), Trail Making Test(11) (TMT), and the Controlled Oral Word Association(12) (COWA). The tests have published normative data that take into account age and, where appropriate, education and gender. NCF, while strongly encouraged, will be optional and with guidance lead by MDA Neuropsychology team. NCF baseline is pre-surgery for the Postop SRS arm and pre-radiation for the Preop SRS arm. Variations from this are to be noted and recorded.

2.7 PATIENT-REPORTED OUTCOMES (PROS) AND HEALTH-RELATED QUALITY OF LIFE (HRQOL)

Radiation therapy to the brain can impact functioning and potentially neurocognitive function. Patient-reported outcome measures (PROs) provide a mechanism to assess the impact.(13) We hypothesize that mean symptom severity and mean symptom interference as well as mean neurologic and cognitive factor score and change score from baseline will be similar in both arms. We also predict that the total symptom severity on the M.D. Anderson Symptom Inventory Brain Tumor (MDASI-BT); the symptom interference subscale; and the specific items of fatigue, neurologic factor items and cognitive factor items score loss will be similar in both arms.

HRQOL will be measured using the EQ-5D-5L, a well-established, validated measure that has been used in brain metastases populations.(14) Symptom assessment measures such as the MDASI-BT have been specifically developed in patients with brain tumors to capture patient self-reports of symptom severity and the patient's perception of the impact or interference with daily

CONFIDENTIAL

activities. The MDASI-BT has demonstrated reliability and validity in the brain tumor patient population, including predictive validity for tumor recurrence.(15, 16) Both the EQ-5D-5L and MDASI-BT are brief and therefore are not a significant burden for patients to complete. Data will be analyzed longitudinally and compared between treatment arms.

3. FEASIBILITY

Prior retrospective studies have performed pre-operative SRS. Resection occurred within 48 hours to 2 weeks. University of Pittsburgh (UPMC) presently has an unreported phase II study of pre-operative SRS with doses similar to those used with RTOG 9005. Our study would be volumetric based consistent with MDACC standard SRS clinical practice.

4. SIGNIFICANCE/ VALUE OF PROSPECTIVE TRIAL

Currently, there are no randomized prospective trials supporting the use of pre-operative SRS to the post-operative SRS to the surgical cavity. Retrospective evidence suggests that pre-operative SRS may decrease LMD rates. Validating pre-operative SRS local control and LMD rates in a prospective randomized fashion can be accomplished at MDACC given the volume of cancer patients with metastatic brain disease treated with both surgery and radiosurgery.

5. PATIENT SELECTION, ELIGIBILITY, AND INELIGIBILITY CRITERIA

5.0 PATIENT SELECTION & ELIGIBILITY

The study will be a controlled prospective randomized trial in which eligible patients will be randomized to one of two arms, conventional surgery followed by stereotactic radiosurgery to the resection bed (Arm 1) or preoperative radiosurgery followed by surgery (Arm 2).

5.1 INCLUSION CRITERIA

5.1.1 Patients must be ≥ 18 years of age

5.1.2 The primary lesion pre-operatively can have a maximum diameter of ≤ 4cm for single fraction and ≤7cm for multifraction therapy.

5.1.3 Patients must be considered candidates for SRS within ± 30 days of surgical resection as defined by either H&P or presentation at Brain metastasis tumor board conference note.

5.1.4 Patients must have a Karnofsky Performance Scores ≥ 60 or ECOG ≤ 2 within 30 days of enrollment.

5.1.5 Patients must agree to randomization as documented by signing the Institutional Review Board (IRB) approved consent form.

5.1.6 No radiographic evidence of leptomeningeal disease on radiology report or neuro-radiologist review

5.1.7 Documented history of malignancy

5.2 EXCLUSION CRITERIA

5.2.1 Patients who have received prior radiation therapy to the brain for parenchymal brain metastases to more than 3 stereotactically treated lesions or lesions closer than 1cm to the target/cavity lesion.

5.2.2 Patients who have received prior whole brain radiation therapy

5.2.3 The primary tumor is small-cell lung cancer, lymphoma, leukemia, or multiple myeloma.

5.2.4 For females, if they are pregnant or breast-feeding (The exclusion is made because gadolinium may be teratogenic in pregnancy).

5.2.5 Patient with prior surgical resection for a brain metastasis

6. REQUIREMENTS FOR STUDY ENTRY, TREATMENT, AND FOLLOW-UP

6.1 PRETREATMENT EVALUATION

We will follow the Office of Clinical Research SOP 04: Informed Consent Process. Written or electronic informed consent will be obtained by the research team after confirming patient eligibility. Informed consent for this study may only be obtained by the Principal Investigator or an assigned designee. This delegation will be included in a protocol delegation log that will be signed by the site's PI.

This study will allow non-English speaking subjects to be enrolled. Verbal Translation Preparative Sheet (VTPS) will be used if a translated consent form is not available in the subject's language. The consent form will be translated into the language of the subject after 2 or more occurrences. This will apply to any MD Anderson and Cancer Network site patients.

Informed Consent Forms for enrolled patients and for patients who are enrolled but later determined not eligible to receive study treatment (screening failures) will be maintained at the

CONFIDENTIAL

study site. . The investigator will maintain a screening log to record details of all patients screened and to confirm eligibility status and which eligibility criteria caused screen failure, as applicable.

Participating Cancer Network sites and Multi-Center participating sites must ensure that the method of obtaining and documenting the informed consent and the contents of the consent must comply with ICH-GCP and all applicable regulatory requirements.

all consented subjects must be registered using OnCore. Any patient not registered in OnCore before treatment begins will be considered ineligible and registration will be denied.

Patients will be enrolled in the study and randomized within 3 research business days (not weekends or holidays) of the informed consent process. Patients randomized to pre-operative SRS first would undergo SRS followed by surgical resection within up to 30 days. Patients randomized to surgical resection first would undergo surgical resection followed by start of post-operative SRS/SRT within up to 30 days. Minor deviations noted if these are within 45 calendar days or 6 weeks. The patient's complete history (including details of prior chemotherapy, radiotherapy, or surgery for systemic disease, and concurrent non-malignant disease), general physical exam, complete neurological exam, and determination of the KPS, will be reviewed. While there is not a limit on number of non-randomized SRS lesions at presentation for enrollment, patients with ≤ 15 SRS lesions are encouraged.

To register a participating Multi-Center or Cancer Network Site patient, the following documents should be uploaded into the institutionally appropriate cloud-based storage system:

- Signed informed consent and documentation of the informed consent process
- HIPAA authorization form (if separate from the informed consent document)
- Completed eligibility checklist with supporting source documents

After the central registration and randomization, an e-mail confirmation of the registration and assigned protocol patient identification number (PPID#) will be sent to the participating Cancer Site's team by MDACC Research team member.

CONFIDENTIAL

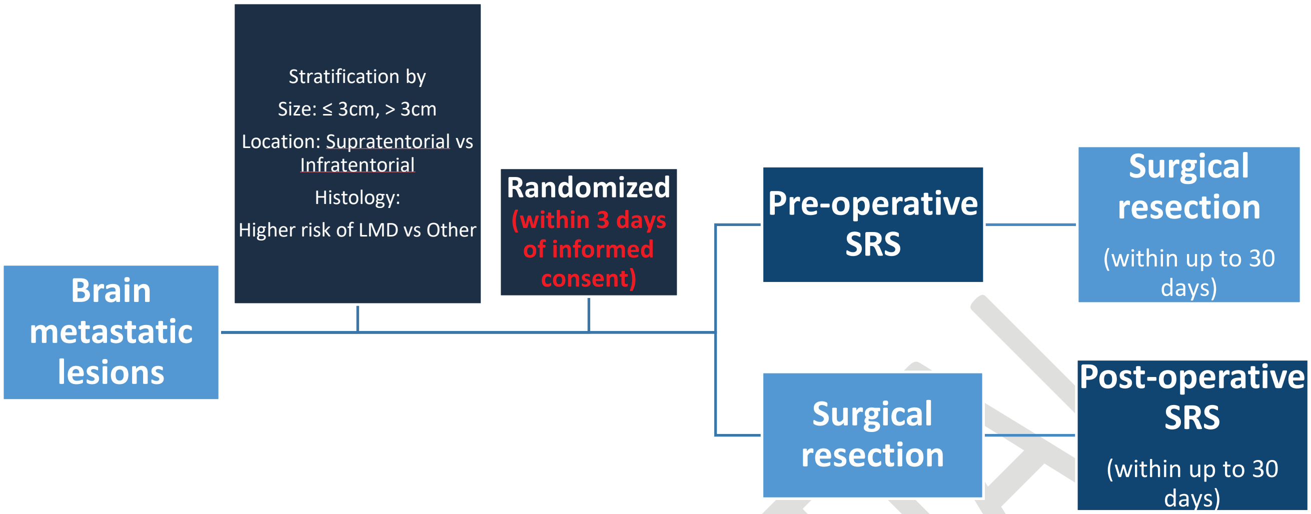

6.2 STRATIFICATION

- Prior to patient enrollment, the investigator should ensure that all of the following stratification requirements are met: The max size of brain lesion: ≤ 3cm, OR > 3cm
- The location: Supratentorial vs Infratentorial
- Histology: histology with presumed higher risk of LMD (i.e. breast, non-small cell lung cancer, and melanoma) versus other histology

6.3 PRE-TREATMENT ASSESSMENTS

**PRE-TREATMENT REQUIRED ASSESSMENTS (standard of care)**	
**Prior to registration and randomization**	
Brain MRI w/ contrast [SOC]	Within 25 business days of prior to enrollment (5 weeks)
Informed consent [Research]	Prior to registration
Documented history of malignancy	Recorded within 5 yrs. prior to registration and randomization
Medical History (physical exam with neurologic when available i.e. can include motor function when feasible for non-telemed visit) [SOC]	Within 25 business days (5 weeks)
Karnofsky performance status or ECOG performance status	Within 25 business days (5 weeks)

CONFIDENTIAL

**Prior to Treatment (Radiation or Surgery, depending on randomization)**	
Brain MRI w/ contrast for planning [SOC]	Within 14 business days (2 weeks) (+7 days allowed)
Urine or Serum (blood) pregnancy test (if female with reproductive organs intact and <55). Documentation must be available for post-menopausal subjects (i.e., no menses for 12 months without an alternative medical cause), women with permanent sterilization (e.g., hysterectomy, bilateral salpingectomy and bilateral oophorectomy, or tubal ligation) and/or age over 55. [SOC]	Within 14 business days (2 weeks) of radiation
Baseline neurocognitive function testing: HVLT-R, TMT, COWA [Research]	Within 30 business days (6 weeks), before RT in Preop SRS arm or before surgery in Postop SRS arm treatment starts
QOL questionnaires: MDASI-BT, EQ-5D-5L [Research]	Within 30 business days (6 weeks), before RT in Preop SRS arm or before surgery in Postop SRS arm treatment starts
Blood Specimen collection [Research]	Within 30 business days (6 weeks),<br>1. before RT in Preop SRS arm and before or at surgery (peri-op)*<br>2. before or at Surgery (peri-op) in Postop SRS arm*

*Please see blood specimen collection in section 12 regarding baselines

** Spine MRI and CSF cytology (Lumbar puncture) recommended if clinically or radiographically suspicious including but not limited to:

- MRI report or Brain Metastasis Tumor Board Conference review of suspicion for LMD (i.e. documented evidence of LMD is excluded)
- Clinical neurological symptoms unexplained by focal lesion concerning for LMD

Tissue pathology is required on protocol as part of SOC surgery (section 11) and residual research blocks are requested within 3 days before surgical date when feasible for the MDACC site.

6.4 ASSESSMENTS IN FOLLOW UP

ASSESSMENTS IN FOLLOW UP	
Assessments (standard of care)	1st year: 45, 120, 180, 270, 360 calendar days from completion of surgery & SRS (+/- 30days)<br>(i.e. 1.5, 4, 6, 9 and 12 months from treatment +/- 4 weeks)<br>2nd year: 15, 18, 21, 24 months (+/- 4 weeks)<br><br>Telemedicine is allowed with review of data by research team and imaging review by MD Anderson Quantitative Imaging Analysis Core (QIAC) or study radiologists.
Physical exam/ Neurologic exam	
Brain MRI w/ contrast	
Karnofsky performance status	
Assessments (research)	
Neurocognitive: HVLT-R, COWA, TMT	1st year: 45, 180, 360 calendar days from completion of surgery & SRS (+/- 30days)<br>(ie.1.5, 6, and 12 months from the start of treatment (+/- 4 weeks)
QOL: MDASI-BT, EQ-5D-5L	1st year: 45, 180, 360 calendar days from completion of surgery & SRS (+/- 30days)<br>(ie.1.5, 6, and 12 months from the start of treatment (+/- 4 weeks)
Blood Specimen collection	1st year: 45, 180, 360 calendar days from completion of surgery & SRS (+/- 30days)<br>(i.e. 1.5, 6, and 12 months from treatment +/- 4 weeks)

CONFIDENTIAL

6.5 STUDY CHART

	**Trial Period**	**Study Screening or at Baseline after randomization**	**On treatment**		**Post Treatment**											
			Pre-op SRS With Surgery	Surgery with post-op SRS												
	**Scheduling Window (months):**				1.5 month		4 month	6 month	9 month		12 month	15 month	18 month	21 month	24 month	
	**Administrative**															
	Brain MRI (SOC)	x			x		x	x	x		x	x	x	x	x	
	Informed Consent	x														
	**Clinical Procedures/ Assessments**															
	History/physical exam with neurologic (if non-telemed) & medical history (SOC)	x														
	Follow up note/neuro (if non-telemed)				x		x	x	x		x	x	x	x	x	
	KPS or ECOG PS	x			x		x	x	x		x	x	x	x	x	
	GPA[f]	x														
	Serum pregnancy test (if applicable primarily before RT as SOC)		x	x												
	**Assessments/ Imaging/Laboratory**															
	Brain MRI for therapy[d] (SOC)	x			x		x	x	x		x	x	x	x	x	
	Spine MRI w/ contrast[c]	x														

	(if warranted for SOC)															
	CSF cytology (Lumbar puncture)[c] (if warranted for SOC)	X[d]														
	Survival Status	X			x		x	x	x		x	x	x	x	x	
	Review of Adverse events (research)				x		x	x	x		x	x	x	x	x	
	Medication(steroid) [e]	x			x		x	x	x		x	x	x	x	x	
	Immunotherapy Targeted therapy Chemotherapy	x			x		x	x	x		x	x	x	x	x	
	Neurocognitive[b]: HVLT-R, TMT, COWA	X (research)			X (researc h)			X (research)			X (research)					
	QOL[b]: MDASI-BT, EQ-5D-5L	X (research)			X (researc h)			X (research)			X (research)					
	Specimen collection: [a]	x (research)			X (blood/ research)			X (research)			X (research)					

Footnotes

(a) Baseline(s), 1.5 month, 6 month and 12 month or at POD with exploratory endpoints to correlative with POD/ development of LMD which can be at other time points Highly encouraged though patients may opt-out. Limited to UT MD Anderson Cancer Patients only.
(b) Highly encouraged though patients may opt-out. MDACC methods will be provided by Neuro-psych team if logistically available at that site for NCF.
(c) Spine MRI and CSF cytology (Lumbar puncture) indicated if clinically suspicious at any time point on study including but not limited to:

i. MRI with focal sulci enhancement
ii. MRI with dural leptomeningeal enhancement
iii. MRI with cerebellar folia linear enhancement
iv. Clinical neurological symptoms unexplained by focal lesion

(d) MRI brains will be evaluated at every point ordered or follow up and at evidence of new lesions, progression or new intracranial treatment for the primary and secondary endpoints
(e) Medication refers to prescriptions. Our study would focus on steroids such as dexamethasone of > 4mg daily equivalent or higher. Our study also considers other steroids such as prednisone (≥27mg) and methylprednisolone (≥22mg) at a certain dose equivalent when given continuously over 2 weeks. Also, our study considers anti-epileptics and neuro-cognitive (e.g. memantine) if records are available.

CONFIDENTIAL

(f) GPA: This value is to be entered only at baseline and is applicable to subjects diagnosed with lung, melanoma, breast, renal cell carcinoma and GI cancers.

Additional Notations:

SOC = standard of care

Medical history refers to prior surgeries to the brain (not for brain mets) and prior SRS (up to 3 lesions) the subject had before signing the informed consent. This will be entered at baseline. Baseline symptoms with review of the AE appendix for the prioritized screening for symptoms prior to enrollment and grade will be included in the study entry note. These are used for comparison to AEs in subsequent post treatment visits.

Other cancer-related therapies refer to chemotherapy, targeted therapy, and immunotherapy taken by the subject during the treatment and follow-up period.

Residual tissue or CSF for SOC will be standardly banked for research purposes. Patients consenting for optional additional bio-specimen collection (blood and/or CSF) may be consented for draws at research intervals above.

CONFIDENTIAL

7. TREATMENT PLAN/REGIMEN DESCRIPTION

7.1 RADIATION THERAPY

Linear accelerator SRS with either circular collimation or mini-multileaf collimation or Gamma Knife (GK) SRS will be allowed.

7.2 DEFINITION OF TARGET VOLUMES AND MARGINS

The target volume for postop SRS may include the surgical cavity with a 1-2mm CTV margin.
For LINAC based therapy a PTV of 1-2mm will be added.
Gamma Knife planning will be per departmental policy.

7.3 DOSE AND DOSE CONSTRAINTS

Doses and prescriptions per MDACC CNS radiation oncology clinical practice guidelines (Appendix H) are encouraged. Non-study target lesions (i.e. non-cavity lesions) can be treated and managed per the treating MDs discretion.

7.4 Immobilization and Simulation

Immobilization

Patients will be immobilized in the supine position using an immobilization device such as a Gamma Knife frame, ICON Gamma Knife mask, or an Aquaplast mask for SRS over the head. Patients will be treated in the immobilization device.

7.5 Simulation Imaging

For LINAC based SRS, a non-contrast treatment-planning CT scan of the entire head region using the smallest possible axial slice thickness not exceeding 2 mm will be required and should match the MRI axial slice thickness as much as possible. The treatment-planning CT scan must be acquired with the patient in the same position and immobilization device as for treatment. This should be obtained within 21 days prior to initiating treatment.

7.6 MRI treatment imaging

Routine MRI brain with and without contrast will be performed including post-contrast volumetric axial-T1-weighted 3D-fast spoiled gradient echo sequence at <1.5 mm slice thickness to permit

reformatting in 3 planes (axial, coronal and sagittal) for treatment planning. MRI for preop SRS planning should be within 10 days and MRI for postop SRS cavity planning should be within 21 days. For diagnostic imaging sequences, sequences may include T1 with and without contrast, T2, FLAIR, DWI and perfusion studies may be obtained routinely. Some conditions such as brain neoplasms with radiation treatment changes can require additional sequences to assess progression of disease versus radiation treatment effect. Imaging used routinely by our radiology department at MDACC includes Advanced Brain Tumor Imaging, such as magnetic resonance spectroscopy (MRS) and MR perfusion (MRP) imaging.

7.7 Patient-specific QA

Patient-specific QA while not required is encouraged and should follow guidelines of CNS Radiation Oncology MDACC Main campus.

For patients enrolled or the first 3 patients regardless of the randomized arm at participating MD Anderson Cancer Network or Multi-institutional locations, the radiation treatment plans will be required to undergo a central review by study P.I (DNY) before beginning protocol-specified radiotherapy. Sites are required to upload the radiation therapy treatment plans into an institutionally approved cloud-based storage folder, including the planned dates of radiation therapy. These treatment plans will be reviewed and approved within 3 business days by the MD Anderson team.

7.8 Daily Treatment Localization/IGRT

Verification orthogonal films, CBCT or images are required. For all forms of IMRT dose delivery, orthogonal films or images that localize the isocenter placement shall be obtained. For Gamma Knife ICON, CBCT can also be used. No additional imaging is required for Gamma Knife frame based treatment.

8. Definition for Leptomeningeal disease

8.1 Radiographic evidence of LMD as reviewed by study neuro-radiologist or MDACC Brain metastasis conference review including: presence of malignant cells in CSF/ positive CSF cytology and/or MRI of brain with LMD and/or LMD enhancement or nodular contrast enhancement of the spine cord on MRI

** Spine MRI and CSF cytology (Lumbar puncture) recommended if clinically suspicious including:

MRI with sulci enhancement

CONFIDENTIAL

MRI with leptomeningeal dural enhancement

MRI with cerebellar folia linear enhancement

Clinical neurological symptoms unexplained by focal lesion

Of note, for both screening and surveillance, if work up for LMD is deemed negative patient may enroll on trial or continue on trial.

9. Off Study Requirements

Once the patient completes the 24 month's post-treatment assessments as described on "study chart" patient will be taken off from protocol imaging surveillance and toxicity follow up on the protocol if not being followed for exploratory aims. All patients will have survival status and study endpoints documented up to at least 2 years prior to off-study (per intention to treat).

Lost to Follow-Up: Patients will be considered lost to follow-up if there is no evidence of clinical documentation (e.g. any clinical interaction) with the patient in the electronic medical record in the past 12 months and the research team has attempted to contact the participant for follow-up at least 3 times with no response. Lost to Follow-Up will be noted in the appropriate source, study database, and the subject will be off protocol imaging surveillance and toxicity follow up. The patient will continue to be followed for survival status until the end of the 24-month post-treatment follow-up period. If contact with the patient is regained, an attempt should be made to restart the post-treatment follow-up assessments (listed in Section 6.5 Study Chart).Patients after they develop LMD or require whole brain radiation therapy (WBRT), will be off disease surveillance and toxicity follow up 1 year after or when they die. If this 1-year period passes the 24-month post-treatment time point, they are on-study for the exploratory aim as listed above. If the 1-year period is complete before the 24-month post-treatment time point, the patient will be followed for survival up to the 24-month post-treatment time point.

Patient who develop new lesions will remain on study and will be managed at the discretion of the treating physician. Distant intracranial recurrences will be documented and recorded.

10. ADVERSE EVENTS REPORTING REQUIREMENTS

Adverse Event (AE) - An adverse event (AE) can be any unfavorable and unintended sign, symptom, or disease temporally associated with the use of an investigational product, whether or not considered related to the investigational product (attribution of unrelated, unlikely, possible,

CONFIDENTIAL

probable, or definite). NCI Common Terminology Criteria for Adverse Events (CTCAE) version 5 will be used to assess acute and late toxicities.

Serious Adverse Events (SAEs) — The descriptions and grading scales found in the NCI Common Terminology Criteria for Adverse Events (CTCAE) version 5.0 will be utilized for SAE reporting. All appropriate treatment areas should have access to a copy of the CTCAE version 5.0. All serious adverse events that occur after the initial treatment, during treatment, or within 30 days of the last radiation dose must be reported to the MDACC Principal Investigator on the local institutional SAE form.

Serious adverse events (SAEs) that meet expedited reporting criteria will be recorded.
Definition of an SAE: Any adverse experience occurring that results in any of the following outcomes:

- Death;
- A life-threatening adverse drug experience;
- Inpatient hospitalization or prolongation of existing hospitalization;
- A persistent or significant disability/incapacity;
- A congenital anomaly/birth defect;
- Important medical events (IME) that may not result in death, be life threatening, or require hospitalization may be considered an SAE, when, based upon medical judgment, they may jeopardize the patient and may require medical or surgical intervention to prevent one of the outcomes listed in the definition.

Requirements for prompt reporting to the IRB

The MD Anderson IRB requires that the investigators submit a prompt report for SAEs that are both serious and unanticipated. The investigators are responsible for providing a meaningful summary, analysis or explanation of the event reported in order to enable the IRB to make further determinations regarding the protection of the participants.

Timeline for prompt reporting to the IRB

1. **Within 1 working day (24 hours) from the time the research team becomes aware of the event =** Deaths that are unanticipated and definitely, probably or possibly related to study intervention (drug or device) that occur during and within 30 days after the last day of active study intervention.

CONFIDENTIAL

2. **Within 5 working days from the time the research team becomes aware of the event =** All other serious, unanticipated, and definitely, probably, or possibly related AEs

11. SPECIMEN COLLECTION

Patients will undergo standard of care tumor resection as part of the study. Tumor and tissue collected after standard of care procedures will be banked for research purposes including for correlation of to the study endpoints. Residual tissue or specimens relating to pathology will be banked or stored in neuropathology under the direction of Neuro-pathology. A portion of banked tissue will be frozen in liquid nitrogen. Banked tissue will be subjected to histopathological and immunohistochemical analysis to assess of extent of cellularity, proliferative potential, necrosis, vascularity, and inflammation. For each of these variables, the specimens will be scored semiquantitatively by a board certified Neuropathologist (JTH). Material will also be assessed for correlations with local control, LMD and intracranial control among others. Molecular and histopathologic changes associated with receiving pre-operative SRS will also be evaluated. Residual biospecimens collected for standard of care including CSF for patients with workup for LMD will be collected and stored for research purposes in research labs. In cases where spinal fluid can be accessed as standard surgical procedure, CSF may be obtained at time of surgery. Banked frozen and FFPE tissue will be subjected to molecular analyses, including but not limited to multiplexed immunofluorescence, TCR sequencing, and single-nucleus RNA-seq.

12. OPTIONAL SPECIMEN SUBMISSIONS

Patients may be offered the opportunity to consent to optional specimen collection. If the patient consents to participate, specimens will be collected for research purposes only and analyzed or banked by research lab for MDACC enrolled patients. Baseline blood would be after enrollment and prior to initial treatment with either surgery (in postop SRS arm) or RT (in preop SRS arm). Blood may be collected close to window if needed with documentation of variation. Postop SRS arm would have blood at baseline peri-operative, 1.5, 6, 12 month. Preop SRS arm would have blood at baseline, peri-operative, 1.5, 6, 12 month. CSF is banked at any points collected in a SOC procedure (i.e. surgery or LMD work up).

Specimen Type	Collection Time Points	Collection Information and Requirements/Instructions for Site

CONFIDENTIAL

Specimen 1:<br><br>1 Streck tubes, 1 CPT	Baseline: Prior to first treatment (SRS or Surgery)<br><br>At each study follow up or tumor progression	Processed Serum aliquots – minimum of 0.5 mL per aliquot in five (5) 1 mL cryovials. Store at -80°C (-70°C to -90°C) until ready to batch ship on Dry ice.<br><br>Storage: -80°C and ship frozen
Specimen 2:<br>Whole Blood for DNA: anticoagulated whole blood in EDTA tube<br>(2 EDTA)	Baseline: Prior to first treatment (SRS or Surgery)<br><br>At each study follow up or tumor progression	Frozen whole blood samples containing 1.0 mL per aliquot in three (3) 2 mL cryovials<br><br>Store at -80°C (-70°C to -90°C) until ready to batch ship on Dry ice.

12.1 Blood Draws:

1. The baseline blood collection will be for testing of germline mutations, serum cytokines, PBMCs, and circulating angiogenic proteins, and blood- based studies.
2. Subsequent blood samples may be used in part to screen for study eligibility for a separate clinical trial if applicable, the investigation of disease evolution, and blood-based biomarkers of disease or response to therapies.

12.2 Subsequent Blood Draw(s) (optional, for research purposes):

- ≤ 40mL of blood will be obtained during surgery to explore tumor microenvironment.
- ≤ 40 mL of blood will be obtained post-SRS or post-operatively, but prior to initiating systemic therapy.
- ≤ 40 mL of blood will be obtained upon completion of therapy at the time of follow up with treating oncologist.

CONFIDENTIAL

- ≤ 40 mL of blood may be obtained at time of progression, as defined by RANO or treating physician's determination, at the time of follow up with treating oncologist.

Baseline/ Signing Consent	At time of Surgery	Post-op or Post-SRS, but prior to initiating systemic therapy	Completion of surgery and SRS therapy	At time of Progression
≤ 40 mL	≤40 mL	≤40 mL	≤40 mL	≤40 mL

12.3 Blood and CSF Samples Collected (included in samples listed above):

Blood Samples

Approximately 30 ml total of peripheral blood will be obtained for research purposes from participants as above or per the Study Calendar:

- Approximately 20 ml will be collected for ctDNA analysis.
- Approximately 10ml will be collected in a standard tube for banking.

All tubes will be labeled with HIPAA compliant participant identifier, and sealed in plastic bags labeled as biohazard. These specimens will be banked in Dr. de Groot's lab for future analysis.

CSF Samples

Approximately 10-20 ml total of CSF will be obtained via LP for research purposes from participants per the Study Calendar in patients who do not have contraindications to LP (ex: high risk of herniation)

- Approximately 10 ml will be collected for ctDNA analysis.
- Approximately 10 ml will be collected in a standard CSF collection tube for banking.

All tubes will be labeled with HIPAA compliant participant identifier, and sealed in plastic bags labeled as biohazard. These specimens will be banked in Dr. de Groot's lab for future analysis.

CONFIDENTIAL

13. EXPLORATORY IMAGING STUDIES (NON TISSUE)

13.1 IMAGING ANALYSIS

13.1.1 RADIOMIC ANALYSIS

Correlative analysis includes assessing radiomic features of metastatic brain lesions receiving SRS. As an exploratory analysis, radiomics will be used to assess features prognostic of response

to radiation. Radiomic features will be correlated with pathology obtained as standard of care from surgical resection.

13.1.2 MULTIPARAMETRIC QUANTITATIVE ANALYSIS

Correlative analysis includes assessing multiparametric images to generate parametric maps of quantitative measures from perfusion and diffusion imaging when obtained. The quantitative measures and parametric maps will be correlated with pathology. Exploratory Imaging Studies are limited to UT MD Anderson Cancer Patients only or patients with OSF whose standard MRIs include these.

14. NEUROCOGNITIVE EVALUATION

14.1 NEUROCOGNITIVE CLINICAL TRIAL BATTERY

The Neurocognitive Clinical Trial Battery is a brief, sensitive, repeatable, highly standardized, objective battery of neurocognitive tests has been demonstrated to be practical in terms of burden on the patient and site, with good compliance in multicenter clinical trials(8, 9, 15). The tests have published normative data that take into account age and, where appropriate, education and gender. As in RTOG 0614, six alternate forms of the HVLT-R and two alternate forms of the COWA will be employed to minimize practice effects.

Patient Reported Outcomes (all patients are required to participate) Symptom Burden (NOTE: Translations not available for this protocol)

Symptom burden will be assessed using the MDASI-BT-modified (Armstrong 2006). The MDASI-BT has demonstrated reliability and validity in the primary brain tumor patient population, including predictive validity for tumor recurrence(15, 16). The MDASI-BT was developed and validated for use in the brain tumor patient population and typically requires less than 4 minutes to complete. It consists of 23 symptoms rated on an 11-point scale (0 to 10) to indicate the presence and severity of the symptom, with 0 being “not present” and 10 being “as bad as you can imagine.” Each symptom is rated at its worst in the last 24 hours. Symptoms included on the instrument are those commonly associated with cancer therapies and those associated with neurologic and cognitive symptoms associated with the tumor itself. The MDASI-BT also includes ratings of how symptoms have interfered with different aspects of the patient’s life in the last 24 hours. These interference items include: general activity, mood, work (includes both work outside the home and

CONFIDENTIAL

housework), relations with other people, walking, and enjoyment of life. The interference items also are measured on 0-10 scales. Neuro-cognitive testing while optional is encouraged for all outpatients and not required for inpatients. If patients cannot attend the in-person assessment, a link to complete the questionnaires online will be sent to those patients via encrypted email using Redcap's survey function or paper if the questionnaires cannot be completed using REDCap's survey function.

14.2 HEALTH RELATED QUALITY OF LIFE

Health related quality of life will be assessed using the EQ-5D-5L. The EQ-5D-5L is a standardized self-report measure of health status developed by the EuroQol Group in order to provide a simple, descriptive profile and a single index value for clinical and economic appraisal (17). The initial EQ-5D was adapted to include a 5-level measure of severity to improve reliability and sensitivity and reduce ceiling effects. It consists of 2 pages, the EQ-5D-5L descriptive (mobility, self-care, usual activities, pain/discomfort, anxiety/depression) using 5 levels (no problems, slight problems, moderate problems, severe problems, and extreme problems) and the EQ Visual Analogue scale (EQ VAS). The EQ VAS records the respondent's self-rated health on a 20 cm vertical, visual analogue scale with endpoints labeled 'the best health you can imagine' and 'the worst health you can imagine', with respondents marking an X to indicate health today and then writing the number marked on the scale in a box below. The digits for 5 dimensions can be combined in a 5-digit number describing the respondent's health state. If patients cannot attend the in-person assessment, a link to complete the questionnaires online will be sent to those patients via encrypted email using Redcap's survey function or paper if the questionnaires cannot be completed using REDCap's survey function.

15. STATISTICAL CONSIDERATIONS

15.1 STATISTICAL ANALYSIS/POWER/STUDY DESIGN:

The 1 year post-op LMD-free rate was approximately 70% in our institutional post-op SRS trial. We wish to increase this to 90%. Randomization will be 1:1. Pocock-Simon will be used to balance the stratification factors of target size, anatomical location, and histology (i.e. histologies with higher risk of LMD such as breast, non-small cell lung cancer, and melanoma versus others). We base our sample size justification on a log rank test comparing freedom from LMD between groups with assumed 1 year rates of 90% vs 70% with 2-sided 5% alpha and 80% power, accrual rate based on prior historical post-op SRS trial (2 per month), and 12 month post accrual follow up

CONFIDENTIAL

(i.e. follow up on all patients after last patient is accrued) requires 110 evaluable patients' total. The final analysis will be performed when 22 LMD events have been observed among the 110 evaluable patients. Taking into consideration updated rates of dropout from the COVID-19 pandemic period, we plan to expand the total accrual to up to 180 patients in order to obtain 110 randomized and evaluable patients assuming if a dropout rate that could be as high as 30-33%. Patient enrollment will stop once a total of 110 evaluable patients is reached or a total of 22 LMD events have been observed, whichever comes first. The expected total study duration will be up to 85 months. A planned interim analysis will be conducted. A planned interim analysis will be conducted when 11 LMD events are observed (50% of required 22 total). The trial will be stopped early for success if $p < 0.003$ and for futility if $p > 0.722$ (Obrien-Fleming boundaries). We will evaluate the LMD rates and dropout rates at this interim analysis and readjust the sample size if our original assumptions turn out to have been wrong. Kaplan-Meier estimates will be utilized for survival.

Aalen-Johansen estimates will be used for LMD, local recurrence and distant brain recurrence. Uni- and multi-variable analysis of time to LMD, time to local recurrence, time to distant brain recurrence will be done using Fine-Gray proportional hazards regression, and for overall survival will be done with Cox proportional hazards regression. Model assumptions will be verified graphically by residual analyses. LMD, local recurrence, and distant brain recurrence are all subject to competing risk bias from death without these events (i.e. death from non-CNS causes). For the sample size calculation, we assume LMD sub-distribution hazard is constant, and that the intervention has little or no effect on death without LMD.

15.2 Stratification

Pocock-Simon will be used to balance the stratification factors. Patients will be stratified by:
Size of the lesion maximum unidimensional approximation: ≤ 3cm, >3cm
Anatomical location: Infratentorial vs Supratentorial
Histology: histology with presumed higher risk of LMD (i.e. breast, non-small cell lung cancer, and melanoma) versus other histology

15.3 Randomization

Patients will be randomized 1:1 to receive pre-operative SRS to post-operative SRS.

Randomization will be performed via the Department of Biostatistics CTC website. East 6 software was used to estimate the required number of patients and compute the group sequential stopping boundaries.

15.4 Total Accrual

The total accrual for this study will be up to 180 patients in order to obtain 110 randomized and evaluable patients. Patient enrollment will stop once a total of 110 randomized and evaluable patients is reached.

15.5 Study Endpoints

Primary Endpoint

The primary endpoint is 1 year LMD-free rate for surgically resectable metastatic brain lesions status post a combination of radiation and surgery.

15.6 Secondary Endpoints

- 1 year local control rate for the study target lesion
- 1 year distant brain control
- 1 year overall survival

15.7 Exploratory Endpoints

- local control of non-target (non-cavity) lesions from time of initial treatment
- intracranial disease control
- Delta radiomics will be used to assess features prognostic of response to radiation
- Bio specimen collection for circulating tumor cell correlation
- Cerebral spinal fluid analysis
- Neurocognitive function, as measured by neurocognitive decline on HVLT-R, COWA, and TMT Parts A and B, and Clinical Trial Battery Composite (CTB COMP) score
- Symptom burden, as measured by the MDASI-BT
- Health outcomes, as measured by the EQ-5D-5L

15.8 Primary Objectives Study Design

Primary Hypothesis and Endpoints

We hypothesize the 1 year LMD-free rate will increase from 70% with post-op SRS to 90% with pre-op SRS (1,2). The outcome from this phase will be a new paradigm for the treatment of brain

CONFIDENTIAL

metastasis. It would decrease the incidence of a fatal neurological state of disease by decreasing the rate of LMD.

15.9 Definitions of Primary Endpoints and How These Will Be Analyzed

First evidence of leptomeningeal disease, defined on at least one of the following assessments: Evidence of LMD as reviewed by study neuro-radiologist or MDACC Brain metastasis conference review including but not limited to the presence of:

Malignant cells in CSF (positive CSF cytology) and/or

MRI of brain with LMD and/or

LMD enhancement or nodular contrast enhancement of the spine cord on MRI

15.10 Analysis of Symptom Burden

Four subscales (symptom severity, symptom interference, neurologic factor, and cognitive factor score) as well as certain individual items (fatigue, neurologic factor items, and cognitive factor items) of the MDASI-BT will be analyzed. For discrete time point analyses, the change from baseline to each follow-up time point (1.5, 6, and 12 months from end of treatment) will be calculated and compared between treatment arms using a t-test or Wilcoxon-Mann-Whitney test, depending on the normality of the data.

Mixed effects models will be used to assess changes of the four subscale scores (symptom severity, symptom interference, neurologic factor, and cognitive factor score) across time using all available data while adjusting for stratification variables and other baseline characteristics. Since missing data is expected, it will be handled in a similar way as described for Neurocognitive Function above.

To assess the prognostic ability of baseline symptom severity, symptom interference, fatigue, neurologic factor items, and neurocognitive factor items on time to neurocognitive decline, the cause-specific Cox proportional hazards regression model will be used.

15.11 Assessment of Quality Adjusted Survival

The VAS and index scores from the EQ-5D-5L will be calculated at each time point (baseline, 1.5, 6, and 12, months from the start of treatment) and compared between treatment arms using a t-test with a 2-sided significance level of 0.05. If there are significant differences, then a cost analysis will be conducted.

CONFIDENTIAL

Quality-adjusted survival (QAS) is defined by the weighted sum of different time episodes added up to a total quality-adjusted survival time. A Markov model will be used to model cost for this analysis. The Medicare reimbursement in dollars/QAS will be calculated as a function of the monetary cost per relative value of each health state and its duration. The EQ-5D-5L index score at 1.5, 6 and 12 months will be used for the cost-utility analysis. The z-test will be used to test the hypothesis that the cost-utility in the two treatment arms is the same with significance level of 0.05. The cost-utility using the Medicare reimbursement in dollars/QAS between the two treatment arms after adjusting for the baseline and stratification variables.

15.12 Analysis of Exploratory endpoint/Radiographic Evaluation

Radiomic texture features and changes in features at diagnosis with pathology and over time will be included in various classifiers for modeling including decision trees, discriminate analysis, and nearest neighbor classifiers using classification learner in Matlab.(18, 19) Lesions defined by the clinical target volume on MRI sequences will be defined in VelocityAI software. Imaging will include MRI T1 with and without contrast, T2 and FLAIR with minimal slices of 5mm skip 6mm (Pre-op STEALTH sequence also allowed.) Standard of care MRI imaging will be analyzed and will include pre-operative imaging, immediate post-operative imaging, initially post SRS imaging, and surveillance imaging available q 2-3 months for 1 year. Imaging processing and feature calculations will be defined by Imaging Biomarker Explorer (IBEX, developed in-house).(20) Texture feature calculations will include intensity histogram, gray level co-occurrence matrix (COM), gray level run matrix (RLM), geometric shape, neighborhood gray tone matrix (NGTM) and histogram of oriented gradients (HOG) on 3D imaging. Collection of these features will be used to evaluate differences in features between pre-op vs post-op therapy including correlation with pathology at diagnosis with directed biopsies and/or whole-mount pathology. Ultimately, determining early progression based on changes in imaging features and using these features to predict outcome would be the goal of building these models. First, a univariate cox regression model will be used to predict progression based on known patient clinical covariates. Next, the significance of each radiomic feature in the model will be calculated to determine whether a model built with only this feature would be a better fit than the null model.(18-20) This will identify features that will be useful for identifying clinically significant radiomics features that will be built upon in iterative models.

16 Data collection (please see ECRF guideline for further details)

CONFIDENTIAL

Demographic information, histology, and GPA (when applicable), and patient symptoms are collected at baseline.

Baseline target cavity lesion (s) – size in 3 dimensions (in cm) at time of enrollment, time of surgery, and time of RT

Baseline target cavity lesion (s) features – hemorrhagic (yes/no); cystic (yes/no); dural involvement (yes/no); ventricular radiographic involvement (yes/no);

Number of other lesions nontarget (unresected lesions) at time of enrollment, time of surgery, and time of RT

RT data (PDF of final treatment plan will be requested):

RT modality: LINAC SRS/fSRT, Gamma Knife, Cyberknife, Tomotherapy, etc.

Target lesions (cavity): treatment modality from above

Non-target lesions (intact unresected lesion): treatment modality from above

Target lesion: prescription radiation dose (total in Gray)

Target lesion: fractionation (total, i.e. 1-5)

Target lesion prescription normalization (typically 45%-99%): enter percentage

Surgical data:

Extent of resection (i.e. Gross total resection vs subtotal resection defined by either NSG or postop MRI);

Resection technique (i.e. enbloc vs piece meal)

Ventricles: Were any ventricles opened during surgery (Yes/No)

Dura: Was tumor adherent to the dura at time of resection

Follow up data:

Number of cavities, number of treated lesions, number of new (RT naïve lesions) at each follow up

MRI and AEs for each follow up period (even if done by telemedicine)

17 Quality Assurance for Cancer Network sites:

17.1 This protocol will adhere to the policies and requirements of Cancer Network Research. The study oversight procedures for study oversight is listed in the Cancer Network Trial Conduct Guidelines.

CONFIDENTIAL

17.2 Central review of MRI images from screening, baseline and follow up will need to be submitted to UT MD Anderson. MRI for screening, at baseline, and at treatment can be sent at the same time within 7 days of the treatment with the exception of the initial QA cases [i.e. section 7.7] which are needed earlier. All other images for follow up will be sent to MD Anderson within 7 days of visit. LifeImage will be used, and sites will be provided a link to upload. Main campus research team will submit images to QIAC for central review.

CONFIDENTIAL

REFERENCES

1. Suki D, Hatiboglu MA, Patel AJ, Weinberg JS, Groves MD, Mahajan A, Sawaya R. Comparative risk of leptomeningeal dissemination of cancer after surgery or stereotactic radiosurgery for a single supratentorial solid tumor metastasis. Neurosurgery. 2009;64(4):664-74; discussion 74-6. doi: 10.1227/01.NEU.0000341535.53720.3E. PubMed PMID: 19197219.
2. Mahajan A, Ahmed S, McAleer MF, Weinberg JS, Li J, Brown P, Settle S, Prabhu SS, Lang FF, Levine N, McGovern S, Sulman E, McCutcheon IE, Azeem S, Cahill D, Tatsui C, Heimberger AB, Ferguson S, Ghia A, Demonte F, Raza S, Guha-Thakurta N, Yang J, Sawaya R, Hess KR, Rao G. Post-operative stereotactic radiosurgery versus observation for completely resected brain metastases: a single-centre, randomised, controlled, phase 3 trial. Lancet Oncol. 2017;18(8):1040-8. Epub 2017/07/04. doi: 10.1016/S1470-2045(17)30414-X. PubMed PMID: 28687375; PubMed Central PMCID: PMCPMC5560102.
3. Suki D, Abouassi H, Patel AJ, Sawaya R, Weinberg JS, Groves MD. Comparative risk of leptomeningeal disease after resection or stereotactic radiosurgery for solid tumor metastasis to the posterior fossa. Journal of neurosurgery. 2008;108(2):248-57. doi: 10.3171/JNS/2008/108/2/0248. PubMed PMID: 18240919.
4. Patel KR, Burri SH, Asher AL, Crocker IR, Fraser RW, Zhang C, Chen Z, Kandula S, Zhong J, Press RH, Olson JJ, Oyesiku NM, Wait SD, Curran WJ, Shu HK, Prabhu RS. Comparing Preoperative With Postoperative Stereotactic Radiosurgery for Resectable Brain Metastases: A Multi-institutional Analysis. Neurosurgery. 2016;79(2):279-85. doi: 10.1227/NEU.0000000000001096. PubMed PMID: 26528673.
5. Patel KR, Burri SH, Boselli D, Symanowski JT, Asher AL, Sumrall A, Fraser RW, Press RH, Zhong J, Cassidy RJ, Olson JJ, Curran WJ, Shu HG, Crocker IR, Prabhu RS. Comparing pre-operative stereotactic radiosurgery (SRS) to post-operative whole brain radiation therapy (WBRT) for resectable brain metastases: a multi-institutional analysis. Journal of neuro-oncology. 2017;131(3):611-8. Epub 2016/12/20. doi: 10.1007/s11060-016-2334-3. PubMed PMID: 28000105.
6. Patchell RA, Tibbs PA, Walsh JW, Dempsey RJ, Maruyama Y, Kryscio RJ, Markesbery WR, Macdonald JS, Young B. A randomized trial of surgery in the treatment of single metastases to the brain. N Engl J Med. 1990;322(8):494-500. doi: 10.1056/NEJM199002223220802. PubMed PMID: 2405271.
7. Patchell RA, Tibbs PA, Regine WF, Dempsey RJ, Mohiuddin M, Kryscio RJ, Markesbery WR, Foon KA, Young B. Postoperative radiotherapy in the treatment of single metastases to the brain: a randomized trial. JAMA. 1998;280(17):1485-9. PubMed PMID: 9809728.
8. Brown PD, Ballman KV, Cerhan JH, Anderson SK, Carrero XW, Whitton AC, Greenspoon J, Parney IF, Laack NNI, Ashman JB, Bahary JP, Hadjipanayis CG, Urbanic JJ, Barker FG, Farace E, Khuntia D, Giannini C, Buckner JC, Galanis E, Roberge D. Postoperative stereotactic radiosurgery compared with whole brain radiotherapy for resected metastatic brain disease (NCCTG N107C/CEC·3): a multicentre, randomised, controlled, phase 3 trial. Lancet Oncol. 2017;18(8):1049-60. Epub 2017/07/04. doi: 10.1016/S1470-2045(17)30441-2. PubMed PMID: 28687377; PubMed Central PMCID: PMCPMC5568757.
9. Meyers CA, Smith JA, Bezjak A, Mehta MP, Liebmann J, Illidge T, Kunkler I, Caudrelier JM, Eisenberg PD, Meerwaldt J, Siemers R, Carrie C, Gaspar LE, Curran W, Phan SC, Miller RA, Renschler MF. Neurocognitive function and progression in patients with brain metastases treated with whole-brain radiation and motexafin gadolinium: results of a randomized phase III trial. J Clin Oncol. 2004;22(1):157-65. doi: 10.1200/JCO.2004.05.128. PubMed PMID: 14701778.
10. Shapiro AM, Benedict RH, Schretlen D, Brandt J. Construct and concurrent validity of the Hopkins Verbal Learning Test-revised. Clin Neuropsychol. 1999;13(3):348-58. doi: 10.1076/clin.13.3.348.1749. PubMed PMID: 10726605.

CONFIDENTIAL

11. Tombaugh TN. Trail Making Test A and B: normative data stratified by age and education. Arch Clin Neuropsychol. 2004;19(2):203-14. doi: 10.1016/S0887-6177(03)00039-8. PubMed PMID: 15010086.
12. Ruff RM, Light RH, Parker SB, Levin HS. Benton Controlled Oral Word Association Test: reliability and updated norms. Arch Clin Neuropsychol. 1996;11(4):329-38. PubMed PMID: 14588937.
13. Gondi V, Paulus R, Bruner DW, Meyers CA, Gore EM, Wolfson A, Werner-Wasik M, Sun AY, Choy H, Movsas B. Decline in tested and self-reported cognitive functioning after prophylactic cranial irradiation for lung cancer: pooled secondary analysis of Radiation Therapy Oncology Group randomized trials 0212 and 0214. Int J Radiat Oncol Biol Phys. 2013;86(4):656-64. doi: 10.1016/j.ijrobp.2013.02.033. PubMed PMID: 23597420; PubMed Central PMCID: PMCPMC4545268.
14. Langley RE, Stephens RJ, Nankivell M, Pugh C, Moore B, Navani N, Wilson P, Faivre-Finn C, Barton R, Parmar MK, Mulvenna PM, Investigators Q. Interim data from the Medical Research Council QUARTZ Trial: does whole brain radiotherapy affect the survival and quality of life of patients with brain metastases from non-small cell lung cancer? Clinical oncology. 2013;25(3):e23-30. doi: 10.1016/j.clon.2012.11.002. PubMed PMID: 23211715.
15. Armstrong TS, Mendoza T, Gning I, Coco C, Cohen MZ, Eriksen L, Hsu MA, Gilbert MR, Cleeland C. Validation of the M.D. Anderson Symptom Inventory Brain Tumor Module (MDASI-BT). Journal of neuro-oncology. 2006;80(1):27-35. doi: 10.1007/s11060-006-9135-z. PubMed PMID: 16598415.
16. Armstrong TS, Vera-Bolanos E, Gning I, Acquaye A, Gilbert MR, Cleeland C, Mendoza T. The impact of symptom interference using the MD Anderson Symptom Inventory-Brain Tumor Module (MDASI-BT) on prediction of recurrence in primary brain tumor patients. Cancer. 2011;117(14):3222-8. doi: 10.1002/cncr.25892. PubMed PMID: 21264841.
17. Janssen MF, Pickard AS, Golicki D, Gudex C, Niewada M, Scalone L, Swinburn P, Busschbach J. Measurement properties of the EQ-5D-5L compared to the EQ-5D-3L across eight patient groups: a multi-country study. Qual Life Res. 2013;22(7):1717-27. doi: 10.1007/s11136-012-0322-4. PubMed PMID: 23184421; PubMed Central PMCID: PMCPMC3764313.
18. Fave X, Zhang L, Yang J, Mackin D, Balter P, Gomez D, Followill D, Jones AK, Stingo F, Liao Z, Mohan R, Court L. Delta-radiomics features for the prediction of patient outcomes in non-small cell lung cancer. Sci Rep. 2017;7(1):588. Epub 2017/04/03. doi: 10.1038/s41598-017-00665-z. PubMed PMID: 28373718; PubMed Central PMCID: PMCPMC5428827.
19. Zhang Z, Yang J, Ho A, Jiang W, Logan J, Wang X, Brown PD, McGovern SL, Guha-Thakurta N, Ferguson SD, Fave X, Zhang L, Mackin D, Court LE, Li J. A predictive model for distinguishing radiation necrosis from tumour progression after gamma knife radiosurgery based on radiomic features from MR images. Eur Radiol. 2017. Epub 2017/11/24. doi: 10.1007/s00330-017-5154-8. PubMed PMID: 29178031.
20. Ger RB, Cardenas CE, Anderson BM, Yang J, Mackin DS, Zhang L, Court LE. Guidelines and Experience Using Imaging Biomarker Explorer (IBEX) for Radiomics. J Vis Exp. 2018(131). Epub 2018/01/08. doi: 10.3791/57132. PubMed PMID: 29364284.

CONFIDENTIAL
