# Supplementary material for: Therapy, Safety, and Logistics of Preoperative vs Postoperative Stereotactic Radiation Therapy: A Preliminary Analysis of a Randomized Clinical Trial
Source: JAMA Oncol. 2025 Jun 18;11(8):890–9. doi: 10.1001/jamaoncol.2025.1770 (PMC12177721; doi:10.1001/jamaoncol.2025.1770)
Supplement: Supplement 2. — eTable 1. Presurgical Procedure Signs or Symptoms by Randomized Group of Preoperative Radiation vs Postoperative Radiation eTable 2. Description of SRT, Radiographic, and Surgical Characteristics of Patients With Brain Metastases Enrolled in the Preoperative vs Postoperative Trial that Completed Both Therapies [file jamaoncol-e251770-s002.pdf]

Supplemental Online Content

Yeboa DN, Li J, Lin R, et al. Therapy, safety, and logistics of preoperative vs postoperative stereotactic radiation therapy: a preliminary analysis of a randomized clinical trial. *JAMA Oncol*. Published online June 18, 2025. doi:10.1001/jamaoncol.2025.1770

eTable 1. Presurgical Procedure Signs or Symptoms by Randomized Group of Preoperative Radiation vs Postoperative Radiation
eTable 2. Description of SRT, Radiographic, and Surgical Characteristics of Patients With Brain Metastases Enrolled in the Preoperative vs Postoperative Trial that Completed Both Therapies

This supplementary material has been provided by the authors to give readers additional information about their work.

© 2025 Yeboa DN et al. *JAMA Oncology*.

eTable 1. Presurgical Procedure Signs or Symptoms by Randomized Group of Preoperative Radiation vs Postoperative Radiation

**Surgical Signs and Symptoms** – n (%)	**Pre-operative (n=45)**	**Post-operative (n=38)**	**P value**
Headache	13 (28.9)	16 (42.1)	0.30
Nausea	4 (8.9)	2 (5.3)	0.68
Seizure	9 (20.0)	7 (18.4)	> 0.99
Mental change	2 (4.4)	3 (7.9)	0.65
Visual problem	7 (15.6)	8 (21.0)	0.71
Sensory change	5 (11.1)	5 (13.2)	> 0.99
Weak motor	14 (31.1)	18 (47.4)	0.19
Confusion	3 (6.7)	5 (13.2)	0.46
Dizziness	6 (13.3)	9 (23.7)	0.35
Speech problem	4 (8.9)	8 (21.0)	0.13
Memory deficit	1 (2.2)	8 (21.0)	0.01
Unsteady gait	6 (13.3)	14 (36.8)	0.02
Cranial involvement	0 (0.0)	0 (0.0)	> 0.99
Other brain*	4 (8.9)	9 (23.7)	0.12

*May include balance changes/fall, hearing issues, agitation or behavioral changes, insomnia, etc.

© 2025 Yeboa DN et al. *JAMA Oncology*.

eTable 2. Description of SRT, Radiographic, and Surgical Characteristics of Patients With Brain Metastases Enrolled in the Preoperative vs Postoperative Trial that Completed Both Therapies

**Characteristics**	**All patients (n = 83)**	**Pre-operative (n = 45)**	**Post-operative (n = 38)**
**Resected lesion Radiographic & Surgical characteristics: Patients with one lesion** – n (%)	73	41	32
Extent of resection			
Gross total	67 (91.8)	38 (92.7)	29 (90.6)
With residual disease	3 (4.1)	2 (4.9)	1 (3.1)
Missing data	3 (4.1)	1 (2.4)	2 (6.3)
Cystic component			
Cystic (> 0cc)	7 (9.6)	3 (7.3)	4 (12.5)
Not cystic	65 (89.0)	38 (92.7)	27 (84.4)
Missing data	1 (1.4)	0 (0.0)	1 (3.1)
Hemorrhagic lesion			
Hemorrhagic	8 (11.0)	6 (14.6)	2 (6.3)
Not Hemorrhagic	63 (86.3)	34 (83.0)	29 (90.6)
Missing data	2 (2.7)	1 (2.4)	1 (3.1)
Necrotic component			
Necrotic (> 0cc)	29 (39.7)	17 (41.5)	12 (37.5)
Not necrotic	43 (58.9)	24 (58.5)	19 (59.4)
Missing data	1 (1.4)	0 (0.0)	1 (3.1)
**Resected lesion Radiographic & Surgical characteristics: Patients with 2 lesions** – n (%)	10	4	6
Extent of resection			
Gross total	9 (90.0)	4 (100.0)	5 (83.3)
With residual disease	1 (10.0)	0 (0.0)	1 (16.7)
Cystic component			
Cystic (>0cc)	0 (0.0)	0 (0.0)	0 (0.0)
Not cystic	10 (100.0)	4 (100.0)	6 (100.0)
Hemorrhagic lesion			
Hemorrhagic	2 (20.0)	0 (0.0)	2 (33.3)
Not Hemorrhagic	8 (80.0)	4 (100.0)	4 (66.7)
Necrotic component			
Necrotic (>0cc)	8 (80.0)	3 (75.0)	5 (83.3)
Not necrotic	2 (20.0)	1 (25.0)	1 (16.7)

© 2025 Yeboa DN et al. *JAMA Oncology*.
