# Supplementary material for: Therapy, Safety, and Logistics of Preoperative vs Postoperative Stereotactic Radiation Therapy: A Preliminary Analysis of a Randomized Clinical Trial
Source: JAMA Oncol. 2025 Jun 18;11(8):890–9. doi: 10.1001/jamaoncol.2025.1770 (PMC12177721; doi:10.1001/jamaoncol.2025.1770)
Supplement: Supplement 3. — Data Sharing Statement [file jamaoncol-e251770-s003.pdf]

Data Sharing Statement

Yeboa. Therapy, Safety, and Logistics of Preoperative vs Postoperative Stereotactic Radiation Therapy. *JAMA Oncol*. Published June 18, 2025. doi:10.1001/jamaoncol.2025.1770

Data
Additional Information: NCT03741673
Data available: No

Additional Information
Explanation for why data not available: Trial is still accruing
